# Abnormal Brain Circuits Characterize Borderline Personality and Mediate the Relationship between Childhood Traumas and Symptoms: A mCCA+jICA and Random Forest Approach

**DOI:** 10.3390/s23052862

**Published:** 2023-03-06

**Authors:** Alessandro Grecucci, Harold Dadomo, Gerardo Salvato, Gaia Lapomarda, Sara Sorella, Irene Messina

**Affiliations:** 1 Clinical and Affective Neuroscience Lab (CL.I.A.N. Lab), Department of Psychology and Cognitive Sciences (DiPSCo), University of Trento, 38068 Rovereto, Italy; 2 Centre for Medical Sciences (CISMed), University of Trento, 38122 Trento, Italy; 3 Unit of Neuroscience, Department of Medicine and Surgery, University of Parma, 43126 Parma, Italy; 4 Department of Brain and Behavioral Sciences, University of Pavia, 27100 Pavia, Italy; 5 Cognitive Neuropsychology Centre, ASST “Grande Ospedale Metropolitano” Niguarda, 20162 Milan, Italy; 6 Milan Centre for Neuroscience (NeuroMI), 20126 Milan, Italy; 7 Department of Psychology, Science Division, New York University of Abu Dhabi, Abu Dhabi P.O. Box 129188, United Arab Emirates; 8 Universitas Mercatorum, 00186 Rome, Italy

**Keywords:** borderline personality disorder, impulsivity, machine learning, data fusion, child trauma, symptoms severity

## Abstract

Borderline personality disorder (BPD) is a severe personality disorder whose neural bases are still unclear. Indeed, previous studies reported inconsistent findings concerning alterations in cortical and subcortical areas. In the present study, we applied for the first time a combination of an unsupervised machine learning approach known as multimodal canonical correlation analysis plus joint independent component analysis (mCCA+jICA), in combination with a supervised machine learning approach known as random forest, to possibly find covarying gray matter and white matter (GM-WM) circuits that separate BPD from controls and that are also predictive of this diagnosis. The first analysis was used to decompose the brain into independent circuits of covarying grey and white matter concentrations. The second method was used to develop a predictive model able to correctly classify new unobserved BPD cases based on one or more circuits derived from the first analysis. To this aim, we analyzed the structural images of patients with BPD and matched healthy controls (HCs). The results showed that two GM-WM covarying circuits, including basal ganglia, amygdala, and portions of the temporal lobes and of the orbitofrontal cortex, correctly classified BPD against HC. Notably, these circuits are affected by specific child traumatic experiences (emotional and physical neglect, and physical abuse) and predict symptoms severity in the interpersonal and impulsivity domains. These results support that BPD is characterized by anomalies in both GM and WM circuits related to early traumatic experiences and specific symptoms.

## 1. Introduction

According to the *Diagnostic and Statistical Manual of Mental Disorders* 5th edition (DSM 5), borderline personality disorder (BPD) is characterized by a pervasive pattern of dysregulation of affect (anger outbursts, depression episodes, and anxiety), cognition (dissociative experiences and self-image disturbances), interpersonal relationships (unstable relationships and fear of abandonment), together with marked impulsivity [1,2]. The disorder has a 3% prevalence in the general population [3,4] and is associated with significant impairment of patients’ psychological functioning [4,5,6].

In the last twenty years, several neuroimaging studies have tried to understand and delineate the neural bases of BPD. So far, neuroanatomical models of BPD indicate significant reduction in grey matter volume and density in the bilateral medial prefrontal cortex (mPFC) [7], medial orbital frontal cortex (OFC) [8,9], bilateral anterior cingulate cortex (ACC) [10], bilateral amygdale, and right parahippocampal gyrus [11,12]. An increase in grey matter volume and density has also been observed in the bilateral precuneus, right medium/paracingulate gyrus, and posterior cingulate gyrus [7]. By contrast, other studies have shown cortical volume reductions using specific regions of interest (ROIs), including in the anterior cingulate cortex, the orbitofrontal cortex, and the right parietal cortex [13,14,15]. Other studies have also examined brain structure in BPD using voxel-based morphometry (VBM) [16] showing structural alterations in the pars opercularis and triangularis of the right inferior frontal gyrus, in the precentral gyrus, and in the right superior frontal gyrus and bilaterally in the temporal lobe cortex [17,18,19]. However, other studies failed to report evidence of volume reductions in the hippocampus or amygdala, see for example [20]. Besides structural alterations, functional alterations have been found too [19,21,22,23].

Based on these results, two distinct (but not necessarily incompatible) neurobiological models of BPD pathology can be outlined. In the first model, the emotional instability/impulsivity/emotion regulation of BPD has been characterized as the core element of this disorder [1,6,8]. In support of this model, functional imaging studies consistently describe a possible phenotype of emotional instability as hyper-reactivity of the amygdala (limbic system) reacting to highly arousing and negative emotional stimuli and impaired recruitment of cognitive processes regulating emotions mainly through activations of the dorsolateral prefrontal cortex [1]; see also [19]. The second explanatory model of BPD underlines the abnormal social and interpersonal functions (including theory of mind deficits) that are characteristic of patients with BPD [17]. Both models (emotion and impulse dysregulation) and (social-interpersonal deficits) may capture different but complementary aspects of BPD symptomatology. Previous studies focused on one of these aspects and returned a partial view of the complexity of BPD. The aim of our studies is to consider both deficits and possibly separate the brain circuits responsible for these disturbances. Besides the conceptual limitation of previous studies, methodological limitations were also present. First, the dependence between the various voxels was not considered, as mass univariate approaches were used [24,25]. This may have limited the possibility to uncover latent patterns of interaction between sparse parts of the brain. Second, the majority of the above-cited studies focused only on specific and a priori decided regions of interest (ROIs), thus limiting the explanatory capacity of the results and possibly hiding the relevance of other structures [26]. As such, these approaches may fail to provide a comprehensive view of a complex disorder such as BPD that may rely on distributed abnormal brain regions.

To overcome the above limitations, a recent study [27] used a data-driven supervised machine learning approach to elucidate whole brain grey matter (GM) alterations in a group of BPD compared with healthy controls (HC), and a clinical control group (bipolar patients). This study showed that a circuit, including basal ganglia, amygdala, and portions of the temporal lobes and of the orbitofrontal cortex, correctly classified BPD against HC (84.62%) and against bipolars (80%). For what concerns the WM contribution, Lapomarda and colleagues [25] applied unsupervised machine learning methods and found that BPD were characterized by white matter (WM) alterations when compared with healthy controls and a clinical control group (bipolar patients) [25]. In particular, BPD patients showed increased white matter concentration in frontal–parietal and temporal regions possibly associated with a dysfunctional top-down emotion regulation [25]. However, in this study, GM and WM were analyzed separately. As such, this analysis does not allow to capture cross-information between modalities [28], as it is not sensitive to find linked hidden structures in the data or any integration between different datatypes [28]. Using both GM and WM properties in a data fusion approach, can greatly improve our understanding of the pathophysiology of BPD [29].

## 2. The Present Study

To overcome the limitations described above, in the present study we used an alternative approach recently developed in the machine learning field that seeks to quantitatively examine the relationship amongst different imaging modalities. This machine learning method is multivariate in nature, thus taking into account the relationship among voxels, is a whole-brain approach, and uses cross-information from multimodal neuroimaging data to explore the complex interplay of brain alterations in different modalities. The method is known as multimodal canonical correlation analysis (mCCA) in conjunction with joint independent component analysis (jICA) [30]. The use of mCCA in conjunction with jICA (mCCA+jICA) [28] is able to fuse two modalities (for example GM and WM) to find the correlations between them (a multimodal canonical covariate matrix, mCCA), and to separate the covariance matrix into independent networks of covarying GM-WM (jICA). As such, mCCA allows a multimodal fusion (MMF) that helps to identify the unique and shared variance associated with each imaging modality that underlies cognitive functioning in healthy controls and impaired mental illness [30,31,32]. Loading coefficients of GM-WM circuits are then tested for their differences between normal and abnormal populations. Previous studies have shown the benefit of MMF for understanding complex syndromes such as schizophrenia [30,31,33] and bipolar disorder [33]. These data-driven parcellation methods can be more biologically plausible than atlas-based parcellations that rely on anatomical or histological properties of the brain tissues that may not be directly related to psychological functions [34]. ICA-based methods by contrast decompose the brain into networks of regions that structurally (or functionally) covary. It is reasonable to assume that if some regions covary in their structural or functional properties across subjects, they do so because they belong to a similar network responsible for similar psychological functions. Thus, the first aim of the present study was to apply a multi-modal fusion (MMF) approach to find covarying GM-WM circuits that differ between BPD patients and HC. We predict to find a GM and WM circuit including temporal and frontal regions, as well as the insula, the amygdala, and the hippocampus in line with previous observations [17,18,19,27]. These regions have been found to be compromised in previous studies [17,18,19,25,27].

Once the covarying GM-WM circuits that differ between BPD and HC have been identified, a supervised machine learning approach known as random forest will be used to build a predictive model of BPD (second aim). This will allow us to test the generalization of our results to new unobserved cases. Random forests will be used to this aim. Random forest is an ensemble learning method for classification based on a forest of multiple decision trees [35,36,37]. The holdout method will be used to train the model on 80% of the participants and to test it on the remaining 20% every time a tree is created. As such, this method can return a measure of generalizability. We predict that the same circuits found in the first aim allow generalization to new cases. Moreover, with random forest being a hierarchical approach (all the networks are estimated and ordered from the best to the worst predictive), it may be that other networks will display a high level of predictability of BPD diagnosis. If the random forest will be able to correctly classify BPD against HC, this means that the networks allowing this classification may be used as potential biomarkers of BPD pathology.

Once the brain networks that separate (aim 1) and predict (aim 2) the BPD diagnosis are found, we aim at characterizing the psychological meaning of these networks. Of note, we aim to find possible relations with childhood traumas that are known to be potential etiological factors of BPD, and with specific symptoms BPD suffer from. Among relevant clinical data that may contribute to the identification of the psychological meaning of relevant neural mechanisms involved in BPD, in the present study we considered traumatic experiences in childhood. Meta-analyses of cross-sectional studies indicated that BPD patients are more likely to report childhood trauma history than nonclinical individuals, including experiences of sexual and physical abuse, neglect, maladaptive parenting, and parental conflict [38], and are more likely to report childhood trauma experiences than other psychiatric groups [39]. Conversely, studies comparing maltreated and non-maltreated children on the prevalence of borderline features show that maltreated children were significantly more likely to present borderline features [40,41]. Prospective longitudinal research provided further evidence for the hypothesis that exposure to adverse events in childhood increased the risk of being diagnosed with BPD in adulthood [42,43,44,45]. In a previous attempt to assess the impact of traumatic experiences on the brain of BPD patients, Dadomo and colleagues [46] reported evidence that a circuit including the amygdala, the Heschl area, the caudate, the putamen, and portions of the cerebellum was predictive of sexual abuse. They also reported that another circuit involving temporal and cerebellar regions was predictive of interpersonal problems. However, this study considered only the contribution of GM, ignoring the fact that early traumatic experiences may affect WM too, and that WM may contribute to specific symptoms. Moreover, that study considered every factor in isolation (independent statistical models), without assessing the contribution of all factors in one unique model to assess for their relative contribution. In the present study, by contrast, we aim at building one unique model, to test a specific hypothesis that early life traumatic events such as physical and emotional abuse or neglect as well as sexual abuse (as measured by the Childhood Traumatic Questionnaire (CTQ) [47]), affect the abnormal brain networks found in aims 1 and 2, and how they in turn support specific symptoms in the cognitive, affective, interpersonal, and impulsivity domains, as measured by the Zanarini Rating Scale for Borderline Personality Disorder (ZAN-BPD) [48]. This analysis may help us in identifying the neural bases of the two models of BPD symptoms discussed before (the emotional-impulsivity dysregulation model vs. the interpersonal-social deficits model). The third aim of the present study was to assess the impact of early traumatic experiences on specific brain circuits, and how such altered brain circuits give rise to specific BPD symptoms according to the current models of this disorder. Mediation analysis will be used to assess the mediating role of the brain networks found in aim 1 and 2 in explaining the link between specific child traumas and specific symptoms. We predict that the networks that differ between BPD and HC are affected by specific traumatic experiences such as emotional and physical mistreatments, and that these abnormal neural circuits explain at least some of the psychological problems BPD patients suffer from such as, for example, interpersonal and impulsivity problems. In summary, we predict that these analyses will shed light on an abnormal impulsivity network and a more interpersonal network.

## 3. Materials and Methods

### 3.1. Participants

A total of 20 patients with BPD (mean age = 35.75 years, SD = 8.61 years) and 45 healthy participants as controls (HC) without history of psychiatric and neurological disease (mean age 36.69 years, SD = 8.46 years), matched for age and sex, were considered. All the data were extracted from the shared OpenNeuro database [49]. Demographic information about participants is displayed in Table 1. The recruitment was carried out by outpatient and support services from around Edinburgh. The exclusion criteria were the presence of neurological disease or mental illness rather than BPD (Structural Clinical Interview, SCID-II, SCID- IV), and the use of psychoactive substance, pregnancy, MRI contraindications. The BPD diagnosis was verified using the *Structured Clinical Interview for Diagnostic Statistical Manual* fourth edition (DSM-IV) (SCID-II). See Table 1.

### 3.2. Questionnaires

To reach our aims, the scores of the Child Trauma Questionnaire (CTQ) [47] that measures five sources of traumas (physical and emotional neglect and abuse, and sexual abuse) and the Zanarini Rating Scale for Borderline Personality Disorder (ZAN-BPD) [48] that measures borderline symptoms in five domains (cognitive, affective, interpersonal, and impulsivity) were taken into consideration. The CTQ is a self-assessment questionnaire developed to evaluate traumatic experiences experienced during childhood that includes five sources of traumas: physical neglect, emotional neglect, physical abuse, emotional abuse, and sexual abuse. The Zan-BPD was designed to capture the severity of symptoms in four main sectors: the Affective sector indicating anger outbursts, feelings of emptiness, and mood instability; the Cognitive sector relative to identity disturbance disassociation and paranoia; the Impulsivity sector relative to impulsivity such as self-mutilative/suicidal efforts; and the Interpersonal sector indicating intense, unstable relationships, and frantic efforts to avoid abandonment.

### 3.3. Preprocessing

T1-weighted images were pre-processed through SPM12 (Statistical Parametric Mapping, https://www.fil.ion.ucl.ac.uk/) [50] and the CAT12 toolbox (Computational Anatomy Toolbox for SPM, http://www.neuro.uni-jena.de/cat/) [51]. After having manually re-oriented all the images, placing the anterior commissure as the origin, the segmentation into gray matter, white matter, and cerebrospinal fluid was computed. The Diffeomorphic Anatomical Registration using Exponential Lie algebra tools for SPM12 (DARTEL) [52] was used for registration. Finally, the normalization to the MNI space with a spatial Gaussian smoothing of 8 was performed.

### 3.4. Data Fusion Unsupervised Machine Learning

mCCA+jICA was applied to structural data using the FusionICA Toolbox (FIT, http://mialab.mrn.org/software/fit) [53] in the MATLAB 2021b environment (https://it.mathworks.com/products/matlab.html) (*MATLAB* (*R2021b*), 2021). The number of components was estimated for both modalities with information theoretic criteria [54]. To assess the consistency of each modality, ICASSO [55,56] was run ten times and the Infomax algorithm was selected. The resulting output consists of a matrix with the number of subjects (rows) and the loading coefficients for each component (columns). Loading coefficients represent how each component is expressed for every subject. Eventual differences between groups was calculated with a *t*-test on the loading coefficients of the GM-WM circuits. As a final step, we converted the independent components into Talairach coordinates in order to specify the brain areas. Areas with both positive and negative values, if present, were considered and plotted in Surf Ice (https://www.nitrc.org/projects/surfice/) using a different template for gray and white matter.

### 3.5. Predictive Model

**Figure 1:**
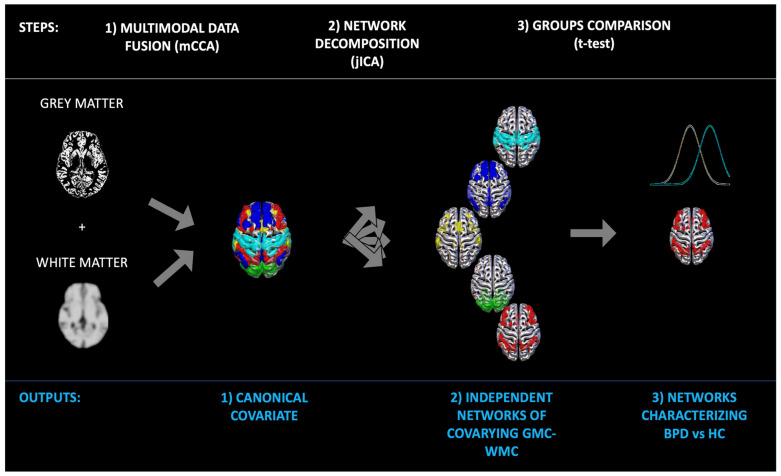
**Schematic workflow of the analyses**. After fusing the two modalities (GM and WM), the brain was decomposed into independent networks of covarying GM-WM (mCCA+jICA). Then Bonferroni corrected *t*-test was used to assess the networks that differed between groups.

**Figure 2:**
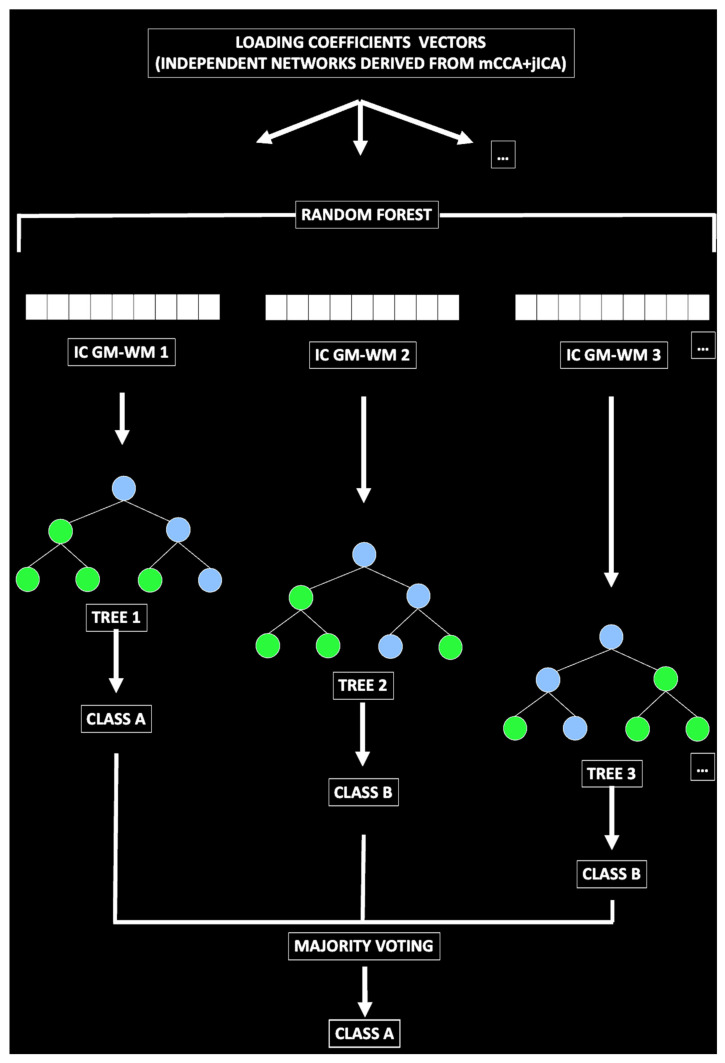
**Predictive model generation**. The loading coefficients of the GM-WM networks derived from mCCA+jICA were entered in a random forest classification model to predict BPD diagnosis. Several trees were generated to classify the labels BPD and HC. Each tree voted for that class. The forest then chose the classification having most of the votes.

Besides testing for differences between groups by using frequentists approaches (e.g., *t*-tests) whose results are limited to the sample considered, we also used a supervised machine learning (SML) approach to extract a statistical model to predict new cases. In other words, we aimed to test our results for their generalization. To carry out this test, an SML method known as random forest classification was used. The name random forest is derived from decision trees, another SML for classification, but it uses multiple trees and then averages their performance (bagging method). To classify a label (e.g., BPD patient) from an input vector (made of the loading coefficients of every independent GM-WM network derived by mCCA+jICA), the input vector is inserted in a tree. The trees classify the label, and then votes for that class. The trees with the lower error rates are the strongest classifiers. The forest chooses the classification having most of the votes [35,36,37]. Random forest is an ensemble learning method for classification based on a forest of decision trees [35,36,37]. For classification tasks, the output of the random forest is the class selected by the most trees. Of note, random decision forests outperform the decision trees (and other SML algorithms) in terms of having less overfitting problems than many other classification algorithms [57]. One of the main reasons random forest was used in this study is that it allows for ranking the importance of variables in a classification problem. In other words, the output is a hierarchical model that allows to estimate the most important feature to the least important. As such, this method can help us understand not only the brain circuits that differ between BPD and HC (frequentist approach), but also to assess which circuits predict new unobserved cases. The algorithm is indeed trained to correctly classify a part of the sample (including BPD and HC), and then tested for its predictive power on the unobserved subsample. The statistical results refer to the prediction of new unobserved cases and as such can be used as a measure of generalization and for the creation of a future possible biomarker [58]. See Figure 1 and Figure 2.

## 4. Results

### 4.1. Groups Comparison

**Figure 3:**
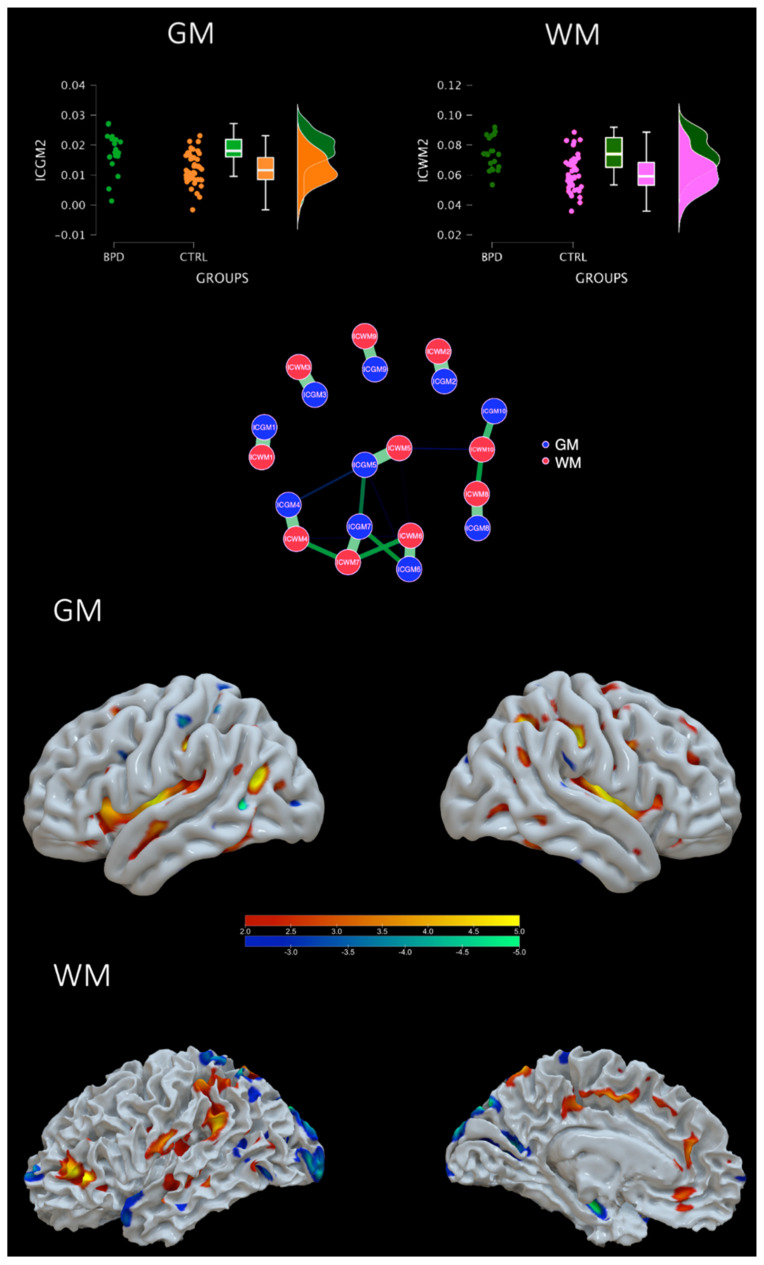
**A covarying GM-WM network that differs from BPD and HC**. Top: violin plots of the loading coefficients for GM and WM of the IC2. Central: network plot showing in green the strength of correlations between components. Bottom: brain plot of positive (increased GM-WM concentration) and negative (decreased GM-WM concentration) of IC2.

The information theoretic criteria estimated 10 independent covarying gray matter (IC-GM) and white matter (IC-WM) networks. The positive values of these networks indicate increased gray/white matter concentration, whereas negative values indicate decreased concentration. The meaning of the covariation between a gray matter and a white matter component refers to a similar pattern of gray/white matter concentration. The results indicate that the following components can be ordered by importance, statistically differed between groups: ICGM2 (t = 3.715, *p* < 0.001) and ICWM2 (t = 4.189, *p* < 0.001), ICGM6 (t = 2.625, *p* = 0.011) and ICWM6 (t = 2.501, *p* = 0.015), ICGM8 (t = −2.384, *p* = 0.020), ICWM8 (t = −2.513, *p* = 0.015), and ICGM4 (−2.335, *p* = 0.023) but not ICWM4 (t = −1.231, *p* = 0.223). Whereas the others did not (ICGM1 *p* = 0.881, ICWM1 *p* = 0.701; ICGM3 *p* = 0.843, ICWM3 *p* = 0.382; IGGM5 *p* = 0.719, ICWM5 *p* = 0.716; ICGM7 *p* = 0.273, ICWM7 *p* = 0.514, ICGM9 *p* = 0.308, ICWM9 *p* = 0.975; and ICM10 *p* = 0.038 ICWM10 *p* = 0.447). However, after applying a Bonferroni corrected threshold (*p* = 0.00125), only the ICGM2-ICWM2 survived. Regions included in the ICGM2 were the post central and precentral gyri, the superior middle temporal gyrus, the insula, the superior, middle, and inferior frontal gyrus, the parietal lobule, the uncus (including the amygdala), cerebellar portions, and the hippocampus among others. See Figure 2 and Table 2A–D. GM and WM of IC2 were also highly correlated with each other (r = 0.857), indicating a strong common profile of alterations in a network that differs between BPD and HC. See Figure 3, network plot, for a representation of each GM-WM component and the strength of the correlation. See Supplementary Table S1.

### 4.2. Predictive Model Results

**Figure 4:**
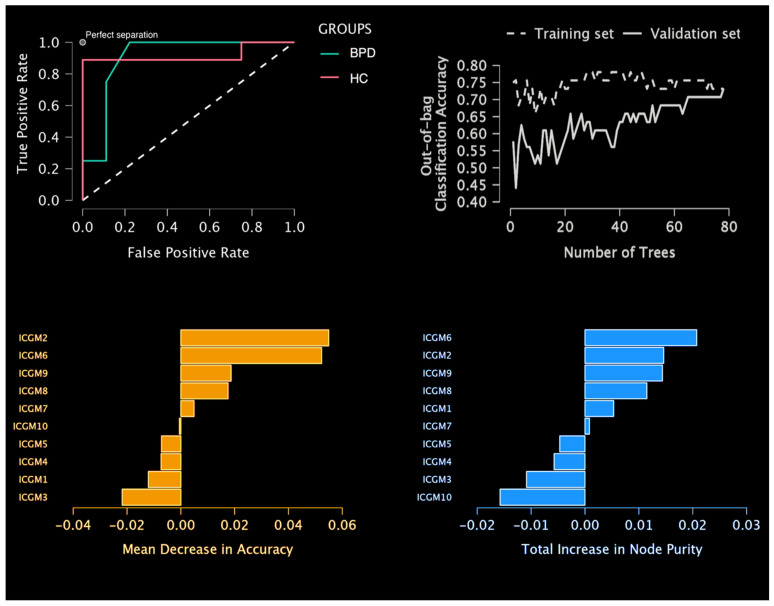
**Prediction of new cases**. Random forest classification performance metrics.

**Figure 5:**
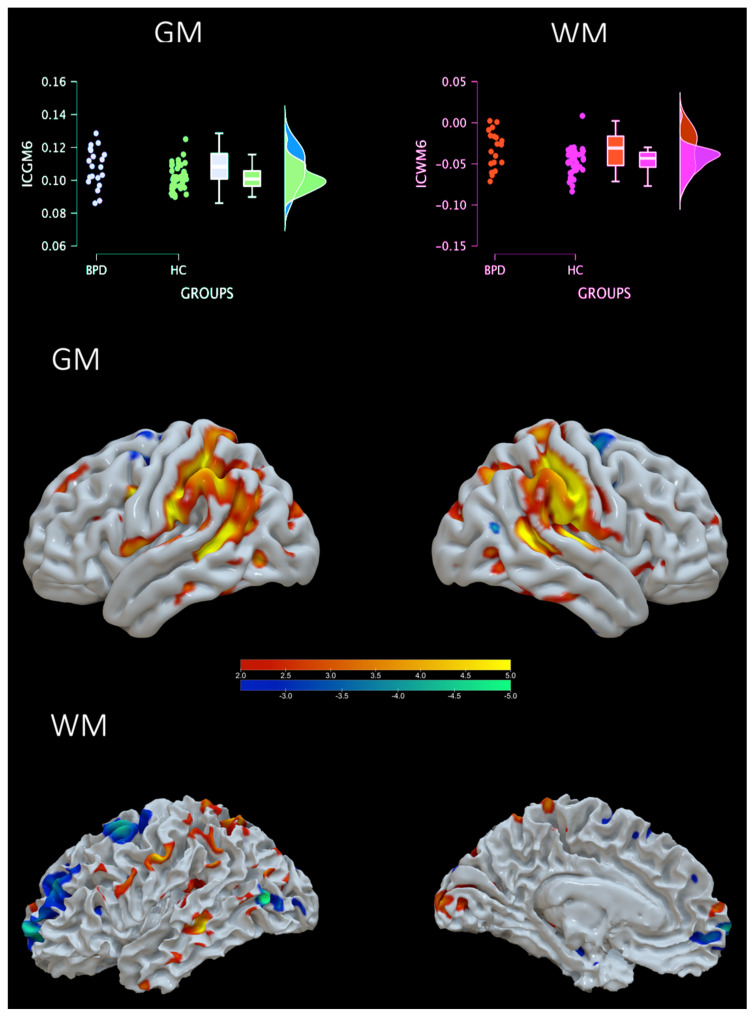
**Brain plots from random forest analysis**. Top: violin plots of the loading coefficients for GM and WM of the IC6. Bottom: brain plot of positive (increased GM - WM concentration) and negative (decreased GM-WM concentration) of IC6.

For the random forest classification, the holdout method of 20% for validation and 20% for testing was selected. In other words, the model was trained on 39 subjects, the model was then validated during learning on 13 subjects, and finally, the model was tested on 13 previously unobserved subjects. The proportion of BPD and HC was kept among the 3 partitions with 30% BPD and 70% HC. The number of trees to reach optimal performance was 78 with 3 features per split. Random forest classification returned a test accuracy of 84.6% for both BPD and HC, a precision (positive predictive value) of 75% for BPD and 88.9% for HC, a recall (true positive rate) of 75% for BPD and 88.9% for HC, a false positive rate of 11.1% for BD and 25% for HC, and a false discovery rate of 25% for BD and 11.1% for HC. The area under the curve was 0.861 for BPD and 0.889 for HC (average = 0.875). The mean decrease in purity confirmed the importance of ICGM2 as a main predictor and main split in trees. Then followed these components: ICGM6, ICGM9, ICGM8, and ICGM7. See Figure 4. Of note, ICGM2 and ICGM6 were very similar in the power of prediction (respectively, a mean decrease in accuracy of 0.055 and 0.052). Building on this result, we also decided to comment further on the ICGM-WM6 component. This component included regions such as temporal–parietal regions, the parahippocampus, the cingulate, the fusiform gyrus, the cuneus, and the insula. The GM and WM of IC6 were also highly correlated with each other (r = 0.777), indicating a strong common profile of alterations in a network that differs between BPD and HC. See Table 3A–D and Figure 5.

### 4.3. Mediation Analysis

**Figure 6:**
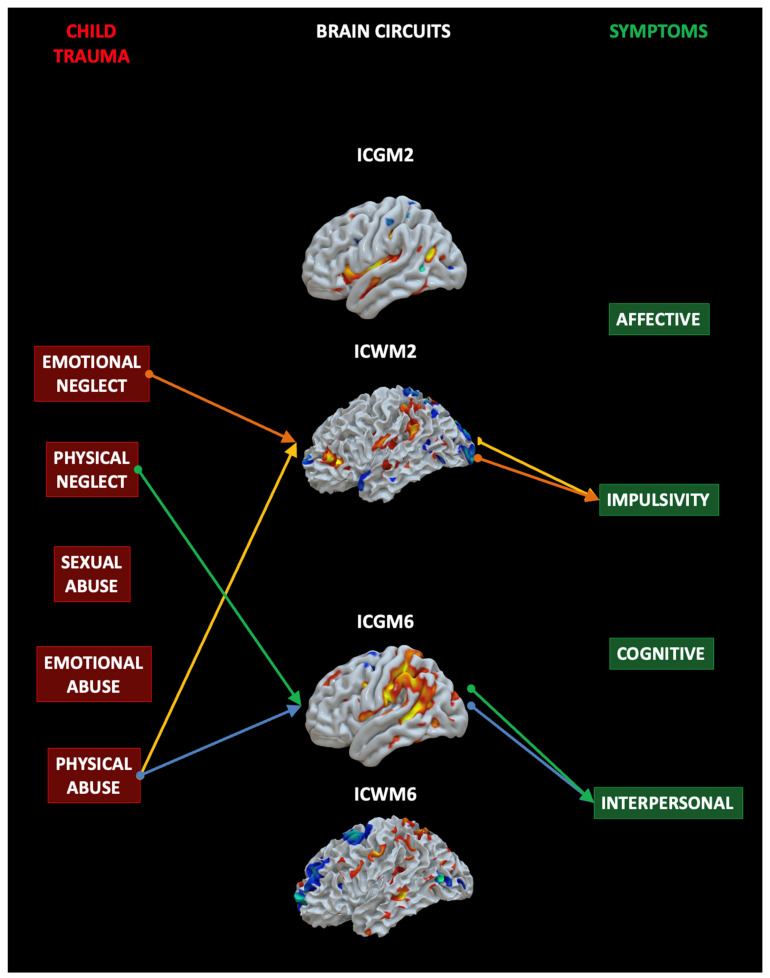
**Mediation analysis results**. Emotional neglect and physical abuse predicted the IC2 (WM) network and that this in turn predicted symptoms in the impulsivity domain. Physical neglect and abuse predicted IC6 (GM) and this in turn predicted interpersonal symptoms. The colors indicate the same indirect effect linking a given child trauma (IV) to a specific symptom (DV) mediated by a specific IC (MV).

To test the hypothesis that early traumatic experiences as measured by the CTQ (independent variables, IVs) may support specific symptoms as measured by the Zanarini BPD symptoms questionnaire (dependent variables, DVs) via the contribution of specific neural circuits that differ between BPD and HC (mediating variables, MVs), we ran a mediation analysis (MA). The MA included the five CTQ subscales (the IVs), the four neural circuits (ICGM2, ICWM2, ICGM6, and ICWM6) (the MAs), and the four symptoms sectors of Zanarini (the DVs). See Figure 6. For what concerns the indirect effect, or the impact of the IVs on the DVs mediated by the MVs of interest for the present study, the results showed that emotional neglect (b = −0.300, 0.018) and physical abuse (b = 0.519, *p* = 0.016) predicted the IC2 (WM) network and that this in turn predicted symptoms in the impulsivity domain. Physical neglect (b = 0.630, *p* = 0.048) and abuse (b = 0.579, *p* = 0.036) predicted IC6 (GM) and this in turn predicted interpersonal symptoms. In summary, at least one of the two modalities of each brain circuit are related to specific traumatic sources and support specific symptoms. See Supplementary Table S2 and Supplementary Figure S1.

## 5. Discussion

In the present study, we contributed to the clarification of the neural basis of BPD by taking advantage of a novel combination of two methodologies (see [59] for a similar method). First, we used an unsupervised data fusion machine learning approach known as mCC+jICA to determine latent abnormal covariance patterns of the gray and white matter that separate BPD patients from healthy controls. *T*-test clarified that one of these networks significantly differs between BPD and controls. Then, we tested the possibility to predict the diagnoses of BPD cases (random forest, a supervised machine learning approach) from these networks. Two networks were found to predict BPD new cases. Last but not least, we tested the role of these circuits as possible mediators between etiological factors of BPD (traumatic experiences in childhood) and different BPD symptoms. We found that the two networks that predict the diagnosis of BPD mediate traumas and impulsivity and interpersonal symptoms. We thus provided support for both of the current models of BPD: the emotional-impulsivity dysregulation model and the interpersonal-social deficits model. In what follows, we describe such patterns in terms of the “impulsivity network” and the “interpersonal network”.

### 5.1. Impulsivity Network

The first network as expected was specifically predictive of BPD symptoms in the impulsivity domain and supports the model of BPD as a disorder of emotional and impulsivity problems. This network included alterations of white matter in several prefrontal regions, including the inferior frontal gyrus (IFG) and extended alterations of gray matter in the insula, postcentral, and precentral gyri. Consistently with our results, all these areas have been previously mentioned as implicated in a variety of impulsivity-related functions [6,60,61], and their structural alterations have been previously reported as associated with individual differences in impulsivity [25,62,63,64,65]. As widely described in “top-down cognitive control” models of impulsivity [66,67], PFC areas may act as a brake on impulsive tendencies by exerting inhibitory control, while subcortical structures propel the occurrence of impulsive behaviors. Among other components of executive control, the role of IFG in impulsivity has been largely investigated due to its role in contrasting impulsivity through the suppression of inappropriate behavioral responses [49]. The insula, instead, appears to be involved in a specific component of impulsivity which concerns the preference of smaller immediate rewards instead of waiting for larger delayed rewards (delay discounting). Due to the relevance of impulsivity-related symptoms in BPD, the alterations in such brain structures were expected from the present study and are in line with previous studies on BPD patients [13,14,15]. Extending such previous studies, the present study suggests that it may be possible to derive an objective biomarker for BPD, generated from impulsivity-related brain structure abnormalities. Of note, these results extend the previous results [25] on structural networks differences between BPD and HC. A previous study [25] provided evidence of a network related to affective disturbances in BPD (correlation with the emotional sector of the Zanarini). Our results add another network to the previous one, a network more related to impulsivity. This may indicate that the model of emotional-impulsivity disturbances in BPD may rely not on one unique network, but rather on two different brain networks, one more related to affective disturbances and another more related to impulsivity.

### 5.2. Interpersonal Network

The second network that was predictive of having a diagnosis of BPD was strictly associated with interpersonal symptoms. This network included different brain regions of increased grey matter concentration in the temporal–parietal junction (TPJ) and other smaller areas located in anterior and posterior midline structures and in the insula. These brain structures correspond to a well-described theory of mind (ToM) network associated with the ability to think about mental states in oneself and especially in others [68]. As such, this network is a good candidate to explain the interpersonal and mentalizing problems these patients suffer from. As part of this network, the TPJ has a core role in inferring the mental states of others [69], as it combines cognitive, social, and affective information during interpersonal situations [69]. Even if most popular neurobiological models of psychopathology are mainly focused on deficits in executive/inhibitory functions, recent contributions have emphasized the additional relevance of semantic functions implicated in the mental representation of self and others [70]. Such semantic alteration is also implicit in clinical models of BPD, which attribute interpersonal difficulties of these patients to impoverished representations of self and others reflected in the shift between positive and negative views of others (e.g., “black-and-white thinking”) [71]. The altered interpersonal-social network (that includes ToM abilities) observed in the present study can be thus considered a significant marker of BPD. Of note, ICGM6 largely overlaps with a network found in a previous study [21]. In that study, the network was found to predict interpersonal problems (measured via the Zanarini questionnaire). Although the method used in our study is very different from the one used in that study (a supervised machine learning method known as multi-kernel regression), the results converge to a similar temporo-frontal network related to interpersonal dysfunctions in BPD patients.

### 5.3. The Impact of Specific Childhood Traumas on the Brain and Symptoms

We also found that the brain circuits correctly classifying BPD were predicted by specific traumatic experiences during childhood (CTQ questionnaire). Specifically, emotional neglect and physical abuse predicted the “impulsivity network”, and physical neglect and abuse predicted the “interpersonal network”. The negative influence of relational traumatic experiences in childhood has been largely theorized in clinical theories about the pathogenesis of BPD [21,25,27]. Previous studies have described the effect of some of these traumatic experiences on the brain of BPD patients [21,71,72]. However, the present study expands our knowledge on this topic by providing a clearer link between specific traumas and separate brain circuits. It is reasonable to think that relationships characterized by physical neglect and abuse can influence the interpersonal and social areas of the brain included in the “interpersonal network” we found. In the same vein, relationships characterized by emotional neglect (but also physical abuse by a violent caregiver) may lead to abnormalities in brain areas responsible for the control of impulsivity. These areas may correspond to the ones that we found in the “impulsivity network”. A previous study reported an association between BPD and sexual abuse [21]. In our study, we could not find this result, probably due to a different method used. It should be noted that the network found in that study [21] includes areas different from the ones that we found in the two networks of our study. Future studies may aim to better explore this issue.

## 6. Conclusions

Our study found two networks of covarying grey and white matter that significantly differ between BPD and HC by using an innovative combination of supervised and unsupervised machine learning approaches. These networks can correctly classify new unobserved cases. Of note, these networks are strictly related to specific child traumas and support symptoms that typically are displayed by borderline patients. Besides the merits, our study does not come without limitations. First, we must acknowledge that the sample size of patients with BPD was quite small. This limitation is common in the scientific literature on BPD as there are a limited number of studies and a few available open datasets. Future studies may aim to use larger samples to replicate these findings. Second, we focused our analyses only on structural brain features. Future studies may aim to explore the contribution of both structural and functional properties. With such a combination, better classification accuracies can be reached and common anatomo-functional alterations can be demonstrated. Besides the limitations, this combined data fusion of an unsupervised and supervised machine learning approach has not been applied previously to understand the BPD brain. We believe that these and other [73] machine learning approaches can be useful in understanding the neural bases of personality disorders and may pave the way for the creation of possible biomarkers.

## Figures and Tables

**Table 1:** Demographic data of the sample.

	BPD	HC	*p*-Values
Participants	20	45	
Age	35.75 (±8.61)	36.69 (±8.46)	*p* = 0.401
Gender	F = 17, M = 3	F = 34, M = 11	*p* = 0.647
Education	≥8 years of formal education		
Exclusion criteria	Neurological disease, psychoactive substance, pregnancy, MRI contraindications, previous head injury	Neurological disease, psychoactive substance, mental illness (SCID-II, SCID-IV), pregnancy, MRI contraindications, previous head injury	

**Table 2:** **Brain areas of IC2**.

Area	Brodmann Area	Volume (cc)	Random Effects: Max Value (x, y, z)
(A) IC2 Increased Grey Matter Concentration			
Postcentral gyrus	2, 3, 40	1.0/1.5	7.4 (−45, −26, 36)/10.0 (43, −24, 39)
Precentral gyrus	13	0.1/0.5	4.2 (−45, −21, 37)/8.7 (46, −21, 37)
Angular gyrus	*	0.0/0.8	0 (0, 0, 0)/8.7 (40, −58, 33)
Sub-gyral	37	1.3/2.0	6.4 (−22, 7, 47)/7.9 (40, −24, 36)
Middle temporal gyrus	19, 21	1.9/0.1	7.9 (−39, −63, 22)/4.0 (58, −45, −2)
Insula	13, 45	3.8/3.8	7.4 (−37, −4, 11)/6.3 (39, −11, 14)
Middle frontal gyrus	6, 8, 11	0.4/1.8	5.4 (−24, 4, 44)/7.1 (25, 17, 41)
Precuneus	7, 31, 39	0.5/2.4	5.9 (−16, −63, 21)/6.6 (13, −61, 38)
Cerebellar tonsil	*	1.2/1.2	6.4 (−27, −44, −42)/6.2 (33, −46, −39)
Superior parietal lobule	7	0.3/0.4	5.9 (−25, −52, 43)/6.3 (30, −55, 43)
Superior frontal gyrus	6	0.4/0.2	6.0 (−22, 11, 48)/4.1 (22, 15, 43)
Pyramis	*	0.4/0.6	4.4 (−9, −80, −23)/5.7 (3, −80, −25)
Inferior parietal lobule	40	0.2/0.7	4.4 (−28, −49, 43)/5.7 (40, −59, 38)
Fusiform gyrus	18, 36, 37	0.5/1.0	3.9 (−48, −42, −21)/5.7 (45, −43, −12)
Uncus (inc amygdala)	20, 28, 36	0.4/0.0	5.6 (−30, −9, −29)/0 (0, 0, 0)
Extra-nuclear	*	0.9/0.1	5.6 (−34, 6, 5)/4.0 (37, −11, 7)
Medial frontal gyrus	*	0.2/0.0	5.6 (−24, 36, 27)/0 (0, 0, 0)
Culmen	*	0.9/0.2	5.4 (−1, −48, −1)/4.9 (3, −48, −1)
Claustrum	*	0.4/0.4	4.8 (−34, −10, 9)/4.4 (36, −4, 6)
Declive	*	0.2/0.5	3.9 (−4, −81, −21)/4.7 (6, −83, −20)
Superior temporal gyrus	39, 41, 42	0.4/0.0	4.6 (−48, −24, 7)/0 (0, 0, 0)
Inferior frontal gyrus	47	0.6/0.1	4.0 (−37, 25, 0)/4.6 (40, 6, 33)
**(B) IC2 Decreased Grey Matter Concentration**			
Posterior cingulate	30, 31	2.3/1.8	13.4 (−22, −58, 8)/11.6 (22, −64, 10)
Cuneus	17, 18, 19, 23, 30	4.0/5.1	11.7 (−16, −69, 10)/13.0 (21, −68, 10)
Extra-nuclear	*	0.6/0.8	11.7 (−21, −53, 8)/10.7 (25, −55, 8)
Thalamus	*	3.8/2.0	9.3 (−10, −17, 9)/5.9 (9, −13, 8)
Lingual gyrus	18, 19	3.7/1.4	9.1 (−18, −52, 5)/6.8 (22, −54, 5)
Lateral ventricle	*	0.3/0.5	6.0 (−28, −58, 8)/8.3 (28, −58, 8)
Middle temporal gyrus	39	0.8/0.3	8.1 (−50, −55, 7)/5.2 (34, −72, 19)
Sub-gyral	*	0.3/1.0	4.7 (−27, −89, 2)/6.8 (28, −54, 5)
Cerebellar tonsil	*	0.0/0.6	0(0, 0, 0)/6.4 (12, −56, −41)
Anterior cingulate	32	0.4/0.6	4.0 (−9, 26, 25)/5.8 (10, 41, 8)
Precuneus	7	0.4/0.1	5.8 (−19, −62, 43)/4.2 (4, −73, 24)
Inferior parietal lobule	40	0.3/0.5	5.7 (−40, −36, 57)/4.4 (53, −26, 28)
Middle frontal gyrus	9	0.6/0.5	5.6 (−37, 15, 38)/5.2 (39, 18, 31)
Postcentral gyrus	1, 2, 3, 40	1.5/0.1	5.6 (−48, −19, 51)/4.1 (64, −28, 21)
Middle occipital gyrus	18, 19	0.4/0.1	5.4 (−28, −86, 4)/3.8 (34, −75, 16)
Inferior semi-lunar lobule	*	0.0/0.6	0 (0, 0, 0)/4.8 (9, −59, −41)
**(C) IC2 Increased White Matter Concentration**			
Middle temporal gyrus	37, 39	0.2/1.2	4.8 (−56, −56, 10)/9.9 (59, −51, −5)
Inferior temporal gyrus	37	0.0/0.6	0 (0, 0, 0)/8.4 (62, −54, −5)
Middle frontal gyrus	6, 8, 9, 10, 11, 46	2.2/3.6	5.5 (−45, 30, 39)/7.6 (49, 34, 33)
Inferior frontal gyrus	10, 46, 47	1.4/0.0	7.1 (−48, 33, −13)/0 (0, 0, 0)
Superior frontal gyrus	6, 8, 9, 10	1.2/1.8	6.5 (−19, 50, 38)/7.0 (22, 0, 65)
Superior temporal gyrus	13, 22, 39	1.0/1.2	4.8 (−52, −16, 9)/6.9 (61, −49, 15)
Claustrum	*	0.3/0.4	5.7 (−34, −6, 6)/6.8 (36, −5, 7)
Insula	13	1.7/1.1	6.5 (−36, −2, 8)/5.8 (36, −5, 11)
Postcentral gyrus	1, 2, 3, 5	1.2/0.3	6.4 (−56, −27, 39)/5.2 (67, −14, 33)
Inferior parietal lobule	40	2.2/1.5	6.0 (−46, −44, 48)/6.3 (52, −50, 43)
Extra-nuclear	*	0.6/0.8	4.6 (−34, −3, 3)/6.2 (36, −1, 7)
Precuneus	7	0.6/0.3	5.7 (−9, −54, 48)/4.8 (22, −63, 46)
Superior parietal lobule	7	0.0/0.4	0 (0, 0, 0)/5.1 (25, −60, 44)
Lentiform nucleus	*	0.6/0.3	4.5 (−15, 3, −5)/4.0 (15, 7, −4)
Cingulate gyrus	23, 24	0.4/0.4	4.3 (−1, 3, 27)/4.5 (3, 1, 28)
Anterior cingulate	24, 32	0.3/0.9	3.7 (−3, 35, 1)/4.3 (3, 35, 1)
**(D) IC2 Decreased White Matter Concentration**			
Inferior parietal lobule	7, 39, 40	0.8/2.7	8.1 (−28, −47, 56)/10.3 (34, −48, 56)
Sub-gyral	7, 20, 40	1.9/2.2	9.6 (−28, −50, 54)/8.0 (31, −44, 51)
Superior parietal lobule	7	1.0/0.6	9.5 (−30, −51, 58)/9.1 (34, −49, 61)
Precuneus	7, 31	1.4/0.7	9.0 (−28, −50, 49)/6.0 (30, −47, 48)
Fusiform gyrus	20, 36, 37	0.4/0.1	7.8 (−40, −17, −24)/4.1 (50, −42, −18)
Cuneus	7, 17, 18, 19, 30	3.6/3.3	6.6 (−10, −76, 31)/6.7 (28, −83, 26)
Middle temporal gyrus	19, 21, 39	0.1/1.3	3.7 (−62, −52, 0)/6.6 (55, −56, 8)
Lingual gyrus	18, 19	1.5/0.8	5.8 (−21, −64, 1)/4.6 (22, −63, 2)
Postcentral gyrus	1, 3, 5	0.8/0.0	5.6 (−43, −30, 62)/−999.0 (0, 0, 0)
Inferior occipital gyrus	18	0.6/0.1	5.6 (−34, −89, −3)/4.2 (48, −80, −2)
Superior frontal gyrus	9, 10, 11	1.2/1.0	5.5 (−15, 65, −10)/5.2 (16, 66, −13)
Inferior temporal gyrus	20	0.5/0.1	5.5 (−43, −17, −27)/4.1 (50, −56, −12)
Middle occipital gyrus	18, 19, 37	0.8/1.9	5.2 (−34, −89, 1)/5.2 (36, −79, 14)
Posterior cingulate	30	0.5/0.7	5.1 (−21, −64, 6)/4.8 (19, −58, 7)

The first column indicates the name of the brain area, the second column indicates the name according to the Brodmann classification, the third column indicates the volume of grey or white matter concentration, and the fourth column indicates the coordinates of the peak for the area. * No Brodmann area was detected by the system.

**Table 3:** Brain areas of IC6.

Area	Brodmann Area	Volume (cc)	Random Effects: Max Value (x, y, z)
(A). ICGM6 Increased Grey Matter Concentration			
Postcentral gyrus	1, 2, 3, 4, 5, 7, 40, 43	6.1/7.4	9.3 (−52, −20, 30)/9.8 (53, −17, 31)
Middle temporal gyrus	21, 22, 39	2.9/1.0	9.7 (−49, −41, 5)/6.2 (50, −43, 9)
Precuneus	7, 19, 39	1.1/2.2	5.0 (−21, −62, 42)/9.0 (30, −61, 35)
Parahippocampal gyrus	19, 36, 37	3.3/2.0	8.7 (−28, −43, −7)/7.7 (30, −44, −5)
Inferior parietal lobule	40	4.2/5.4	5.9 (−55, −23, 30)/8.7 (55, −28, 26)
Precentral gyrus	4, 6, 9, 13, 43	0.9/2.2	6.0 (−36, 4, 29)/8.4 (50, −17, 34)
Superior temporal gyrus	22, 39, 41, 42	3.4/3.4	8.2 (−48, −49, 13)/8.1 (53, −45, 12)
Superior parietal lobule	7	0.4/1.0	5.9 (−22, −59, 44)/8.2 (27, −58, 43)
Sub-gyral	43	1.6/3.1	7.0 (−46, −43, 5)/8.1 (30, −64, 32)
Inferior frontal gyrus	9	0.5/0.0	7.8 (−39, 4, 32)/0 (0, 0, 0)
Fusiform gyrus	19, 20, 37	1.2/0.6	7.6 (−30, −36, −12)/5.6 (28, −47, −8)
Insula	13, 41	0.1/1.9	3.8 (−46, −15, 12)/7.1 (46, −22, 16)
Angular gyrus	39	0.9/0.0	6.5 (−42, −58, 32)/0 (0, 0, 0)
Transverse temporal gyrus	41, 42	0.1/0.7	3.9 (−53, −14, 12)/6.4 (48, −21, 12)
Supramarginal gyrus	40	1.3/0.2	6.4 (−39, −53, 27)/4.8 (52, −48, 22)
Anterior cingulate	24, 32	1.3/0.4	6.3 (−3, 32, −7)/4.9 (3, 29, −10)
Cuneus	18, 19	0.2/1.3	4.0 (−7, −79, 14)/5.1 (10, −88, 14)
Culmen	*	1.3/2.6	4.7 (−22, −41, −12)/4.9 (31, −39, −22)
Cingulate gyrus	31	0.9/0.3	4.7 (−1, −32, 36)/4.4 (1, −32, 39)
Lingual gyrus	18, 19	0.8/0.1	4.6 (−18, −55, −2)/3.6 (22, −53, −2)
Declive	*	0.8/0.0	4.1 (−18, −59, −13)/0 (0, 0, 0)
**(B). ICGM6 Decreased Grey Matter Concentration**			
Sub-gyral	6	0.8/0.8	6.4 (−21, 4, 51)/5.2 (25, −6, 56)
Middle temporal gyrus	*	0.1/0.4	3.8 (−48, −63, 2)/5.8 (42, −66, 16)
Middle frontal gyrus	6, 8	0.9/1.9	4.7 (−33, −2, 43)/5.6 (28, −6, 53)
Fusiform gyrus	20	0.1/0.5	4.0 (−42, −33, −16)/5.0 (43, −27, −18)
Medial frontal gyrus	6	0.6/0.2	4.9 (−18, 7, 51)/4.5 (10, −10, 61)
Superior frontal gyrus	6	0.5/0.3	4.4 (−13, −11, 63)/4.1 (18, −7, 63)
**(C). ICWM6 Increased White Matter Concentration**			
Middle frontal gyrus	9, 46	1.4/0.1	10.2 (−46, 31, 33)/4.1 (48, 39, 26)
Precuneus	7, 19	2.8/2.0	5.8 (−22, −74, 41)/9.9 (24, −70, 49)
Superior parietal lobule	7	0.2/0.8	4.8 (−39, −57, 51)/8.8 (25, −69, 45)
Inferior parietal lobule	39, 40	1.2/4.4	6.2 (−62, −40, 24)/8.4 (49, −42, 56)
Middle occipital gyrus	19	0.9/0.0	7.3 (−39, −85, 17)/0 (0, 0, 0)
Precentral gyrus	4, 6, 44	0.8/4.1	5.8 (−61, −6, 21)/7.3 (55, −17, 35)
Supramarginal gyrus	40	0.7/0.2	7.1 (−62, −42, 27)/4.5 (49, −36, 34)
Postcentral gyrus	1, 2, 3, 7, 40, 43	2.0/3.9	5.6 (−43, −29, 53)/7.1 (53, −16, 31)
Superior temporal gyrus	21, 22, 41, 42	0.4/2.2	5.0 (−65, −40, 21)/7.0 (67, −27, 15)
Cuneus	17, 19	0.4/0.6	4.2 (−7, −92, 3)/6.1 (31, −82, 30)
Sub-gyral	*	0.7/0.3	6.1 (−46, −36, −13)/5.0 (18, −45, 62)
Transverse temporal gyrus	41	0.0/0.4	0 (0, 0, 0)/5.9 (48, −29, 12)
Insula	13	0.0/0.6	0 (0, 0, 0)/5.8 (52, −30, 18)
Middle temporal gyrus	19, 21, 39	0.8/0.3	5.7 (−50, −75, 19)/5.3 (49, −63, 21)
Lingual gyrus	17, 19	0.8/0.0	5.1 (−22, −64, 1)/0 (0, 0, 0)
Superior frontal gyrus	6, 8, 9, 10	0.4/0.1	4.6 (−22, 46, 39)/3.7 (12, 14, 50)
**(D). ICWM6 Decreased White Matter Concentration**			
Precentral gyrus	6, 9	1.3/0.1	8.1 (−37, −6, 62)/3.7 (39, 16, 35)
Superior frontal gyrus	6, 8, 9, 10, 11	5.1/3.0	7.3 (−10, −4, 67)/8.0 (25, −1, 66)
Middle frontal gyrus	6, 8, 9, 10, 46	7.7/2.6	7.6 (−34, −3, 62)/7.0 (31, −3, 61)
Middle occipital gyrus	19, 37	0.4/0.1	6.5 (−49, −67, 5)/3.8 (27, −70, 6)
Superior parietal lobule	7	0.4/0.0	6.4 (−28, −54, 61)/0 (0, 0, 0)
Medial frontal gyrus	6, 11	1.3/0.7	5.2 (−13, −4, 61)/6.4 (15, 1, 58)
Middle temporal gyrus	21, 37, 39	0.2/0.6	5.5 (−46, −64, 6)/5.2 (68, −32, −7)
Sub-gyral	6	1.6/0.3	4.6 (−25, 15, 41)/5.3 (18, −2, 57)
Insula	13	0.4/0.4	4.5 (−36, −5, 10)/4.6 (37, 0, 8)
Cingulate gyrus	32	0.3/0.5	3.8 (−18, 8, 44)/4.4 (15, 16, 36)

The first column indicates the name of the brain area, the second column indicates the name according to the Brodmann classification, the third column indicates the volume of grey or white matter concentration, and the fourth column indicates the coordinates of the peak for the area. * No Brodmann area was detected by the system.

## Data Availability

Publicly available datasets were analyzed in this study. This data can be found at the UCLA Consortium for Neuropsychiatric Phenomics OpenNeuro database, accession number ds000030.

## Supplementary Materials

The following supporting information can be downloaded at: https://www.mdpi.com/article/10.3390/s23052862/s1, Table S1: Other independent components. Table S2: Mediation analysis. Figure S1: Path plot.
