# Supplementary material for: Abnormal Brain Circuits Characterize Borderline Personality and Mediate the Relationship between Childhood Traumas and Symptoms: A mCCA+jICA and Random Forest Approach
Source: Sensors (Basel). 2023 Mar 6;23(5):2862. doi: 10.3390/s23052862 (PMC10006907; doi:10.3390/s23052862)

Supplementary Table S1, T1: OTHER INDEPENDENT COMPONENTS

ICGM1

Area	Brodmann Area	volume (cc)	random effects: Max Value (x, y, z)
Inferior Temporal Gyrus	20, 21	3.8/1.3	11.7 (-40, -13, -30)/8.1 (39, -13, -30)
Fusiform Gyrus	20, 36, 37	3.1/1.3	10.4 (-40, -19, -24)/8.8 (42, -27, -18)
Sub-Gyral	6, 20	3.4/3.1	9.8 (-42, -21, -22)/8.7 (42, -23, -19)
Uncus	20, 28, 36, 38	1.7/2.0	9.7 (-37, -13, -27)/7.7 (36, -10, -32)
*	*	0.1/0.1	4.0 (-4, -84, -26)/4.9 (22, 1, -37)
Precuneus	7, 31	1.1/0.9	9.3 (-15, -59, 22)/6.6 (15, -58, 32)
Middle Temporal Gyrus	20, 21, 37, 38, 39	7.9/4.0	7.2 (-39, -7, -30)/6.0 (50, -56, 10)
Superior Temporal Gyrus	21, 22, 38, 39	4.7/2.9	6.2 (-45, -53, 25)/7.1 (48, -53, 10)
Parahippocampal Gyrus	30, 36	1.1/0.9	7.1 (-39, -29, -19)/6.1 (39, -29, -20)
Middle Occipital Gyrus	18, 19	0.0/1.9	-999.0 (0, 0, 0)/6.7 (39, -72, 16)
Pyramis	*	0.6/0.0	6.5 (-10, -81, -24)/-999.0 (0, 0, 0)
Posterior Cingulate	30, 31	1.1/0.0	6.2 (-12, -59, 15)/-999.0 (0, 0, 0)
Extra-Nuclear	*	0.4/0.1	6.1 (-21, -55, 8)/3.6 (21, -54, 19)
Uvula	*	0.6/0.0	6.0 (-13, -78, -24)/-999.0 (0, 0, 0)
Angular Gyrus	*	0.0/0.1	-999.0 (0, 0, 0)/5.8 (37, -55, 37)
Declive	*	0.6/0.0	5.7 (-7, -81, -21)/-999.0 (0, 0, 0)
Inferior Parietal Lobule	40	0.4/0.1	5.5 (-39, -36, 38)/4.3 (39, -59, 39)
Supramarginal Gyrus	*	0.4/0.1	5.3 (-48, -53, 22)/4.4 (37, -52, 34)
Cingulate Gyrus	31	0.0/0.1	-999.0 (0, 0, 0)/5.1 (16, -55, 29)
Lingual Gyrus	18, 19	0.4/0.3	4.7 (-19, -55, 4)/5.0 (18, -73, 0)
Postcentral Gyrus	3	0.3/0.0	4.7 (-55, -18, 23)/-999.0 (0, 0, 0)
Middle Frontal Gyrus	*	0.0/0.1	-999.0 (0, 0, 0)/4.6 (24, -5, 50)
Cuneus	18	0.1/0.1	4.4 (-7, -86, 20)/3.8 (10, -88, 20)
Anterior Cingulate	24	0.2/0.1	3.9 (-1, 33, -2)/4.3 (1, 32, -4)
Precentral Gyrus	6, 9	0.0/0.1	-999.0 (0, 0, 0)/4.2 (53, -2, 10)
Superior Frontal Gyrus	8	0.0/0.1	-999.0 (0, 0, 0)/3.6 (22, 17, 45)

ICGM3

Area	Brodmann Area	volume (cc)	random effects: Max Value (x, y, z)
**Middle Temporal Gyrus**	**19, 20, 21, 39**	**2.6/1.7**	**9.1 (-48, -41, 5)/6.0 (43, -56, 22)**
**Angular Gyrus**	*	**0.1/0.4**	**4.5 (-42, -70, 30)/8.8 (43, -57, 30)**
**Cuneus**	**7, 18, 19**	**1.3/1.5**	**6.8 (-10, -74, 29)/8.0 (16, -71, 33)**
**Superior Temporal Gyrus**	**21, 22, 39, 41**	**0.3/2.3**	**5.6 (-52, -40, 6)/7.8 (46, -37, 9)**
**Middle Occipital Gyrus**	**18**	**1.4/0.3**	**7.8 (-22, -85, 17)/4.4 (24, -85, 21)**
**Precuneus**	**7, 31**	**1.1/2.1**	**5.7 (-13, -71, 28)/7.2 (16, -68, 30)**
**Declive**	*	**1.2/1.9**	**7.0 (-33, -57, -11)/6.1 (27, -78, -15)**
**Cingulate Gyrus**	**24, 31, 32**	**1.2/0.6**	**6.9 (-4, -50, 26)/5.2 (13, -27, 39)**
**Inferior Semi-Lunar Lobule**	*	**0.3/0.2**	**5.1 (-3, -59, -42)/6.4 (1, -59, -36)**
**Sub-Gyral**	**7, 40**	**1.5/1.2**	**6.3 (-48, -36, 3)/5.6 (56, -47, -6)**
**Posterior Cingulate**	**23, 30, 31**	**1.2/0.1**	**6.2 (-4, -50, 22)/4.3 (3, -47, 23)**
**Medial Frontal Gyrus**	**10, 11**	**1.7/0.4**	**6.1 (-4, 47, -14)/4.6 (12, 48, 3)**
**Fusiform Gyrus**	**19, 20, 37**	**1.1/0.8**	**5.4 (-49, -26, -23)/5.9 (21, -81, -14)**
**Cerebellar Tonsil**	*	**0.3/0.4**	**5.1 (-1, -59, -34)/5.9 (3, -57, -32)**
**Lateral Ventricle**	*	**0.3/0.1**	**5.9 (-25, -8, -19)/4.6 (25, -7, -20)**
*	*	**0.1/0.2**	**3.5 (-34, -50, -13)/4.7 (1, 41, -16)**
**Lingual Gyrus**	**18**	**0.3/1.5**	**5.1 (-19, -70, -2)/5.7 (16, -82, -12)**
**Inferior Frontal Gyrus**	**9**	**0.3/0.0**	**5.7 (-39, 6, 27)/-999.0 (0, 0, 0)**
**Precentral Gyrus**	**6, 9**	**0.1/0.1**	**4.1 (-36, 3, 27)/5.5 (36, 22, 35)**
**Parahippocampal Gyrus**	**28, 34**	**2.2/0.3**	**5.5 (-22, -11, -18)/4.3 (25, -4, -23)**
**Nodule**	*	**0.1/0.5**	**4.0 (0, -51, -30)/5.5 (3, -54, -30)**
**Supramarginal Gyrus**	*	**0.0/0.3**	**-999.0 (0, 0, 0)/5.0 (48, -57, 33)**
**Uncus**	**28**	**0.5/0.3**	**4.9 (-25, -5, -21)/4.2 (28, -4, -19)**
**Inferior Temporal Gyrus**	**20, 37**	**0.6/0.2**	**4.8 (-59, -36, -15)/4.2 (56, -53, -11)**
**Uvula of Vermis**	*	**0.0/0.1**	**-999.0 (0, 0, 0)/4.8 (1, -61, -34)**
**Superior Parietal Lobule**	**7**	**0.2/0.2**	**4.5 (-33, -58, 54)/4.7 (25, -52, 45)**
**Fourth Ventricle**	*	**0.0/0.1**	**-999.0 (0, 0, 0)/4.7 (1, -52, -26)**
**Culmen**	*	**0.3/0.0**	**4.3 (-28, -53, -15)/-999.0 (0, 0, 0)**
**Extra-Nuclear**	*	**0.1/0.0**	**4.2 (-21, -36, 7)/-999.0 (0, 0, 0)**

**Inferior Occipital Gyrus**	**19**	**0.1/0.1**	**4.2 (-39, -73, -5)/3.5 (30, -84, -12)**
**Superior Frontal Gyrus**	*	**0.1/0.1**	**3.8 (-24, 54, -5)/4.2 (19, 56, -12)**
**Anterior Cingulate**	**24, 32**	**0.1/0.2**	**4.0 (-7, 25, 25)/3.8 (4, 30, 12)**
**Middle Frontal Gyrus**	**8**	**0.1/0.1**	**3.8 (-28, 47, 10)/3.9 (34, 19, 38)**
**Uvula**	*	**0.0/0.1**	**-999.0 (0, 0, 0)/3.8 (6, -60, -32)**
**Pyramis**	*	**0.1/0.0**	**3.5 (-34, -66, -31)/-999.0 (0, 0, 0)**

ICGM4

**Area**	**Brodmann Area**	**volume (cc)**	**random effects: Max Value (x, y, z)**
**Thalamus**	*	**7.0/6.2**	**19.7 (-10, -20, 9)/14.9 (12, -18, 9)**
**Extra-Nuclear**	*	**1.3/0.7**	**9.6 (-1, -17, 9)/7.7 (3, -19, 5)**
**Supramarginal Gyrus**	**40**	**0.3/0.8**	**5.2 (-36, -45, 37)/8.7 (43, -54, 29)**
**Cingulate Gyrus**	**23, 24, 31, 32**	**3.5/2.8**	**8.3 (-9, -43, 37)/6.9 (10, -45, 38)**
**Superior Temporal Gyrus**	**38, 39**	**0.0/0.6**	**-999.0 (0, 0, 0)/7.8 (46, -57, 29)**
**Cerebellar Tonsil**	*	**0.5/2.4**	**5.5 (-40, -45, -32)/7.6 (42, -47, -33)**
**Middle Frontal Gyrus**	**6, 8, 9**	**1.2/1.0**	**7.2 (-24, 21, 39)/5.4 (42, 1, 43)**
**Postcentral Gyrus**	**2, 3, 4**	**1.0/0.0**	**7.2 (-52, -22, 31)/-999.0 (0, 0, 0)**
**Precuneus**	**7, 31**	**1.9/0.4**	**7.1 (-7, -48, 30)/5.1 (13, -45, 35)**
**Superior Parietal Lobule**	**7**	**0.3/0.1**	**7.0 (-25, -52, 45)/4.1 (33, -47, 51)**
**Lentiform Nucleus**	*	**0.8/1.4**	**5.1 (-28, -11, 3)/6.7 (28, -9, 5)**
**Culmen**	*	**0.4/0.9**	**4.6 (-42, -48, -29)/6.7 (43, -47, -29)**
**Sub-Gyral**	**6, 8**	**2.2/0.3**	**6.2 (-33, -42, 37)/4.4 (28, -2, 53)**
*	*	**0.1/0.2**	**4.1 (-4, -83, -22)/4.8 (33, -50, -14)**
**Third Ventricle**	*	**0.2/0.3**	**5.9 (-1, -15, 1)/6.0 (1, -12, 1)**
**Angular Gyrus**	*	**0.0/0.4**	**-999.0 (0, 0, 0)/5.9 (43, -57, 32)**
**Inferior Parietal Lobule**	**40**	**1.8/0.4**	**5.6 (-36, -42, 39)/4.6 (50, -36, 40)**
**Declive**	*	**0.3/0.4**	**5.5 (-7, -83, -20)/4.3 (33, -55, -14)**
**Middle Temporal Gyrus**	**19, 39**	**0.1/0.3**	**3.6 (-40, -58, 15)/5.5 (43, -60, 29)**
**Tuber**	*	**0.1/0.8**	**3.7 (-49, -52, -29)/5.5 (49, -54, -29)**
**Lateral Ventricle**	*	**0.4/0.1**	**5.4 (-7, -20, 18)/3.5 (3, -9, 14)**

**Posterior Cingulate**	**23, 30**	**0.4/0.3**	**5.0 (-3, -48, 23)/4.7 (4, -50, 25)**
**Superior Frontal Gyrus**	**6, 8, 9, 10**	**0.5/0.1**	**5.0 (-22, 10, 48)/3.5 (24, 41, 32)**
**Paracentral Lobule**	**5**	**0.2/0.0**	**4.8 (-1, -41, 56)/-999.0 (0, 0, 0)**
**Precentral Gyrus**	**6**	**0.1/0.1**	**3.8 (-46, -21, 37)/4.6 (46, 1, 43)**
**Inferior Temporal Gyrus**	**20**	**0.2/0.0**	**4.4 (-53, -29, -21)/-999.0 (0, 0, 0)**
**Insula**	*	**0.2/0.0**	**4.2 (-45, -35, 21)/-999.0 (0, 0, 0)**
**Pyramis**	*	**0.1/0.0**	**4.2 (-7, -86, -22)/-999.0 (0, 0, 0)**
**Fusiform Gyrus**	**20, 37**	**0.1/0.1**	**3.9 (-50, -32, -20)/4.0 (33, -46, -13)**
**Parahippocampal Gyrus**	**30**	**0.1/0.0**	**3.8 (-15, -48, 4)/-999.0 (0, 0, 0)**
**Inferior Semi-Lunar Lobule**	*	**0.0/0.1**	**-999.0 (0, 0, 0)/3.8 (15, -69, -41)**
**Lingual Gyrus**	*	**0.1/0.0**	**3.6 (-27, -73, -4)/-999.0 (0, 0, 0)**

ICGM5

**Area**	**Brodmann Area**	**volume (cc)**	**random effects: Max Value (x, y, z)**
**Inferior Semi-Lunar Lobule**	*	**5.8/4.9**	**11.2 (0, -57, -38)/10.2 (3, -60, -37)**
*	*	**0.1/0.3**	**3.8 (-12, -67, -4)/6.8 (1, -54, -26)**
**Cerebellar Tonsil**	*	**5.5/4.2**	**10.3 (0, -54, -35)/9.9 (3, -57, -35)**
**Uvula of Vermis**	*	**0.3/0.3**	**9.4 (0, -64, -33)/8.7 (3, -60, -32)**
**Fourth Ventricle**	*	**0.1/0.2**	**4.0 (-1, -52, -24)/9.3 (0, -53, -31)**
**Nodule**	*	**0.6/0.7**	**8.2 (-1, -55, -29)/8.7 (3, -55, -30)**
**Uvula**	*	**1.5/1.9**	**7.8 (-3, -67, -33)/7.4 (7, -69, -33)**
**Inferior Frontal Gyrus**	**9**	**0.5/0.3**	**7.3 (-37, 7, 27)/5.5 (40, 10, 26)**
**Pyramis of Vermis**	*	**0.3/0.1**	**7.2 (0, -70, -28)/6.5 (3, -73, -28)**
**Middle Frontal Gyrus**	**6, 9**	**1.1/0.2**	**6.5 (-36, 12, 27)/7.1 (37, 13, 26)**
**Pyramis**	*	**3.5/2.6**	**7.0 (-15, -79, -33)/6.9 (21, -77, -34)**
**Tuber of Vermis**	*	**0.3/0.1**	**5.8 (-3, -70, -26)/6.6 (3, -70, -26)**
**Uncus**	**28, 34, 36, 38**	**1.0/1.5**	**6.0 (-22, -5, -21)/6.4 (21, -3, -24)**
**Parahippocampal Gyrus**	**28, 34, 35**	**2.0/1.6**	**6.0 (-28, -14, -17)/5.3 (30, -14, -17)**
**Tuber**	*	**1.4/0.6**	**5.8 (-22, -82, -30)/5.6 (19, -83, -30)**
**Superior Temporal Gyrus**	**22, 38, 39**	**0.5/0.8**	**4.9 (-28, 11, -35)/5.8 (42, -56, 17)**
**Sub-Gyral**	*	**0.6/1.2**	**5.4 (-40, 7, 25)/5.8 (34, 16, 25)**
**Precuneus**	**31**	**0.3/0.3**	**4.2 (-13, -71, 28)/5.1 (28, -58, 36)**

**Lateral Ventricle**	*	0.1/0.2	4.4 (-28, -7, -21)/5.0 (25, -8, -20)
**Declive**	*	2.2/2.3	5.0 (0, -65, -14)/4.6 (10, -83, -21)
**Middle Temporal Gyrus**	19, 38, 39	0.4/0.4	4.6 (-43, -59, 14)/4.9 (45, -59, 17)
**Lingual Gyrus**	17, 18	1.8/0.2	4.9 (-4, -91, -7)/3.9 (12, -73, -3)
**Culmen**	*	0.9/0.9	4.6 (-9, -68, -10)/4.9 (1, -48, -4)
**Precentral Gyrus**	4, 6	0.4/0.0	4.8 (-34, 4, 27)/-999.0 (0, 0, 0)
**Middle Occipital Gyrus**	*	0.3/0.1	4.6 (-36, -73, -8)/4.1 (31, -78, 14)
**Inferior Temporal Gyrus**	19	0.1/0.0	4.5 (-49, -58, -2)/-999.0 (0, 0, 0)
**Declive of Vermis**	*	0.1/0.2	3.8 (0, -71, -14)/4.4 (0, -67, -21)
**Fusiform Gyrus**	19	0.3/0.0	4.2 (-34, -73, -11)/-999.0 (0, 0, 0)
**Cerebellar Lingual**	*	0.1/0.1	3.8 (-3, -44, -12)/3.9 (0, -47, -15)
**Inferior Parietal Lobule**	*	0.0/0.1	-999.0 (0, 0, 0)/3.8 (37, -43, 39)
**Cuneus**	7, 18	0.2/0.0	3.7 (-13, -73, 33)/-999.0 (0, 0, 0)
**Superior Frontal Gyrus**	*	0.1/0.0	3.6 (-31, -9, 63)/-999.0 (0, 0, 0)

ICGM7

**Area**	**Brodmann Area**	**volume (cc)**	**random effects: Max Value (x, y, z)**
**Fusiform Gyrus**	20, 36, 37	1.5/1.1	6.4 (-42, -31, -17)/7.4 (42, -30, -17)
**Caudate**	*	1.0/1.7	5.2 (-12, 15, 2)/7.0 (13, 18, 1)
**Superior Temporal Gyrus**	38, 41	1.6/0.6	6.4 (-40, -31, 14)/4.8 (27, 11, -22)
**Sub-Gyral**	6, 20	2.0/1.5	5.5 (-39, 40, 4)/6.2 (42, -23, -20)
**Extra-Nuclear**	47	0.3/1.0	3.8 (-19, -52, 7)/6.0 (24, -53, 8)
**Thalamus**	*	0.6/0.5	5.3 (-3, -10, 6)/5.8 (1, -10, 5)
**Inferior Parietal Lobule**	40	0.1/0.3	3.7 (-46, -37, 23)/5.7 (36, -51, 38)
*	*	0.2/0.4	3.7 (-16, 5, -18)/4.3 (1, 27, -18)
**Anterior Cingulate**	24, 32, 33	2.6/2.8	4.9 (-4, 33, 20)/5.7 (7, 42, -2)
**Middle Frontal Gyrus**	6, 9, 10, 46, 47	2.8/1.5	5.6 (-39, 4, 41)/5.0 (45, 41, -3)
**Insula**	13	2.6/1.3	5.5 (-42, -28, 17)/4.9 (37, -24, 18)
**Parahippocampal Gyrus**	30, 36	0.1/0.5	5.1 (-39, -33, -20)/5.5 (39, -32, -20)
**Inferior Frontal Gyrus**	10, 46, 47	2.6/3.7	5.1 (-16, 9, -17)/5.4 (21, 8, -18)
**Cuneus**	23	0.4/0.1	5.4 (-18, -68, 9)/4.1 (21, -67, 10)
**Middle Temporal Gyrus**	21, 39	1.4/1.8	5.2 (-61, -37, -6)/5.1 (61, -35, -8)

**Medial Frontal Gyrus**	**9, 10, 11, 25**	**1.9/3.1**	**5.1 (-1, 28, -15)/5.2 (3, 47, 13)**
**Transverse Temporal Gyrus**	**41**	**0.3/0.0**	**5.1 (-43, -31, 11)/-999.0 (0, 0, 0)**
**Posterior Cingulate**	**30**	**0.2/0.4**	**4.9 (-21, -65, 9)/5.1 (22, -62, 10)**
**Declive**	*	**0.6/0.0**	**4.9 (-46, -55, -20)/-999.0 (0, 0, 0)**
**Superior Frontal Gyrus**	**9**	**0.7/0.1**	**4.9 (-24, 35, 30)/4.3 (27, 45, 21)**
**Uncus**	**20, 28**	**0.1/0.1**	**4.9 (-25, 9, -22)/4.2 (24, 6, -20)**
**Inferior Temporal Gyrus**	**20**	**0.1/0.3**	**3.6 (-55, -16, -22)/4.7 (39, -13, -27)**
**Lateral Ventricle**	*	**0.1/0.1**	**4.5 (-1, -7, 3)/4.7 (27, -51, 8)**
**Culmen**	*	**0.6/0.0**	**4.5 (-46, -51, -21)/-999.0 (0, 0, 0)**
**Middle Occipital Gyrus**	**19**	**0.1/0.1**	**3.9 (-30, -79, 21)/4.5 (43, -66, -4)**
**Lentiform Nucleus**	*	**0.1/0.1**	**4.2 (-15, 14, -5)/4.3 (16, 13, -3)**
**Supramarginal Gyrus**	*	**0.0/0.1**	**-999.0 (0, 0, 0)/4.3 (37, -48, 36)**
**Precentral Gyrus**	**6, 9**	**0.1/0.3**	**3.6 (-53, 15, 6)/4.3 (40, 8, 37)**
**Cingulate Gyrus**	**32**	**1.2/0.1**	**4.3 (-3, 23, 28)/3.6 (3, 29, 28)**
**Claustrum**	*	**0.1/0.0**	**4.2 (-37, -17, 2)/-999.0 (0, 0, 0)**
**Subcallosal Gyrus**	*	**0.1/0.3**	**4.1 (-13, 8, -14)/4.0 (3, 24, -13)**
**Third Ventricle**	*	**0.0/0.1**	**-999.0 (0, 0, 0)/4.0 (0, -10, 1)**
**Uvula**	*	**0.1/0.0**	**3.9 (-22, -76, -26)/-999.0 (0, 0, 0)**
**Angular Gyrus**	*	**0.0/0.1**	**-999.0 (0, 0, 0)/3.9 (37, -54, 36)**
**Tuber**	*	**0.1/0.0**	**3.9 (-49, -48, -22)/-999.0 (0, 0, 0)**
**Pyramis**	*	**0.1/0.0**	**3.8 (-27, -74, -28)/-999.0 (0, 0, 0)**
**Lingual Gyrus**	*	**0.0/0.1**	**-999.0 (0, 0, 0)/3.8 (27, -85, -3)**
**Inferior Occipital Gyrus**	*	**0.1/0.0**	**3.8 (-40, -69, -3)/-999.0 (0, 0, 0)**
**Rectal Gyrus**	**11**	**0.0/0.1**	**-999.0 (0, 0, 0)/3.5 (4, 40, -23)**

ICGM8

**Area**	**Brodmann Area**	**volume (cc)**	**random effects: Max Value (x, y, z)**
**Supramarginal Gyrus**	**40**	**0.6/0.3**	**9.6 (-40, -54, 26)/4.0 (50, -48, 23)**
**Superior Temporal Gyrus**	**13, 22, 39, 41**	**1.9/3.0**	**8.8 (-40, -57, 29)/8.8 (48, -42, 15)**
**Extra-Nuclear**	*	**2.0/1.9**	**7.2 (-1, -16, 6)/6.7 (15, 17, -3)**
**Caudate**	*	**2.4/2.9**	**7.6 (-13, 4, 15)/8.2 (13, 18, 1)**
**Middle Temporal Gyrus**	**21, 22, 39**	**1.0/0.2**	**8.1 (-48, -45, 9)/4.9 (48, -42, 9)**

**Thalamus**	*	2.6/1.3	8.1 (-4, -13, 6)/7.7 (3, -9, 5)
**Lentiform Nucleus**	*	3.1/2.7	8.0 (-28, -11, 3)/8.1 (28, -9, 3)
**Third Ventricle**	*	0.0/0.3	-999.0 (0, 0, 0)/7.3 (0, -9, 2)
**Culmen**	*	2.9/0.0	7.0 (-33, -40, -21)/-999.0 (0, 0, 0)
*	*	0.1/0.0	4.3 (-27, -37, -18)/-999.0 (0, 0, 0)
**Fusiform Gyrus**	20, 37	0.8/0.0	6.3 (-36, -40, -18)/-999.0 (0, 0, 0)
**Angular Gyrus**	*	0.4/0.0	6.1 (-43, -58, 32)/-999.0 (0, 0, 0)
**Inferior Parietal Lobule**	*	0.1/0.3	4.0 (-43, -48, 23)/6.0 (45, -48, 23)
**Inferior Temporal Gyrus**	20	0.3/0.0	5.7 (-39, -12, -30)/-999.0 (0, 0, 0)
**Insula**	13	0.0/0.1	-999.0 (0, 0, 0)/5.2 (48, -41, 20)
**Sub-Gyral**	*	0.6/0.4	4.2 (-27, -73, 24)/5.1 (42, -45, 23)
**Cuneus**	*	0.0/0.1	-999.0 (0, 0, 0)/4.9 (22, -79, 23)
**Anterior Cingulate**	*	0.0/0.1	-999.0 (0, 0, 0)/4.9 (13, 22, -4)
**Pyramis**	*	0.0/0.3	-999.0 (0, 0, 0)/4.8 (10, -83, -26)
**Postcentral Gyrus**	2, 3	0.1/0.9	3.8 (-62, -15, 24)/4.8 (45, -24, 40)
**Middle Occipital Gyrus**	19	0.2/0.0	4.4 (-40, -67, 3)/-999.0 (0, 0, 0)
**Lingual Gyrus**	18	0.1/0.1	4.4 (-22, -73, -3)/3.8 (30, -73, -4)
**Declive**	*	0.4/0.1	4.0 (-28, -55, -15)/4.2 (10, -84, -22)
**Inferior Semi-Lunar Lobule**	*	0.5/0.2	4.2 (-13, -73, -38)/4.1 (12, -75, -35)
**Precentral Gyrus**	4, 6	0.2/0.2	3.7 (-42, -14, 35)/4.1 (62, -9, 24)
**Uvula**	*	0.0/0.3	-999.0 (0, 0, 0)/4.1 (13, -86, -25)
**Medial Frontal Gyrus**	6	0.0/0.1	-999.0 (0, 0, 0)/4.1 (9, -9, 52)
**Lateral Ventricle**	*	0.1/0.1	3.8 (-13, 0, 22)/3.7 (15, 25, -1)
**Precuneus**	*	0.1/0.0	3.7 (-22, -59, 39)/-999.0 (0, 0, 0)
**Cingulate Gyrus**	31	0.0/0.1	-999.0 (0, 0, 0)/3.7 (13, -26, 39)

ICGM9

**Area**	**Brodmann Area**	**volume (cc)**	**random effects: Max Value (x, y, z)**
**Middle Temporal Gyrus**	19, 20, 39	2.2/1.3	7.7 (-56, -38, -8)/6.6 (50, -55, 0)
**Inferior Temporal Gyrus**	20, 37	0.4/0.4	7.5 (-40, -70, 1)/5.1 (48, -42, -16)
**Middle Occipital Gyrus**	*	1.2/0.1	7.1 (-36, -75, 15)/3.6 (43, -67, -4)
**Culmen**	*	6.3/3.6	7.0 (-33, -49, -18)/6.9 (34, -39, -27)

**Sub-Gyral**	*	1.0/0.3	6.6 (-40, -67, -2)/4.6 (37, -62, -10)
**Fusiform Gyrus**	20, 36, 37	1.1/1.7	6.3 (-36, -46, -18)/6.0 (40, -43, -18)
**Cerebellar Tonsil**	*	1.5/0.4	6.2 (-21, -57, -40)/4.8 (21, -59, -41)
*	*	0.5/0.1	5.5 (-34, -52, -13)/4.6 (36, -49, -16)
**Declive**	*	1.2/0.3	6.1 (-31, -55, -12)/4.0 (33, -56, -14)
**Inferior Semi-Lunar Lobule**	*	0.4/0.4	5.9 (-18, -60, -40)/5.2 (18, -62, -40)
**Uncus**	20, 28, 36, 38	0.6/0.1	5.3 (-25, -2, -38)/3.6 (24, 6, -20)
**Tuber**	*	1.2/0.0	5.2 (-45, -55, -25)/-999.0 (0, 0, 0)
**Inferior Occipital Gyrus**	17, 18	0.3/0.0	5.2 (-40, -67, -5)/-999.0 (0, 0, 0)
**Thalamus**	*	0.8/0.7	5.0 (-10, -14, 9)/4.5 (10, -14, 8)
**Superior Temporal Gyrus**	22, 38, 39, 41, 42	0.2/0.6	4.8 (-45, -60, 18)/5.0 (42, -56, 15)
**Caudate**	*	1.0/0.6	5.0 (-13, 11, 12)/4.6 (16, -5, 21)
**Inferior Parietal Lobule**	40	0.1/0.1	4.7 (-37, -55, 39)/4.6 (37, -35, 38)
**Inferior Frontal Gyrus**	13, 45, 47	0.2/0.4	4.6 (-22, 14, -21)/4.4 (42, 21, 4)
**Insula**	13	0.0/0.6	-999.0 (0, 0, 0)/4.6 (40, -27, 17)
**Parahippocampal Gyrus**	19	0.4/0.2	4.5 (-25, -30, -7)/4.2 (37, -34, -20)
**Lingual Gyrus**	17	0.5/0.1	4.4 (-7, -91, -7)/3.9 (16, -68, 0)
**Anterior Cingulate**	24, 32, 33	0.3/0.4	4.4 (-9, 41, 6)/4.4 (6, 31, 9)
**Precuneus**	7, 31	0.4/0.1	4.3 (-10, -45, 37)/3.6 (9, -48, 37)
**Extra-Nuclear**	*	0.2/0.0	4.1 (-15, -5, 20)/-999.0 (0, 0, 0)
**Transverse Temporal Gyrus**	41	0.0/0.1	-999.0 (0, 0, 0)/4.0 (50, -21, 12)
**Supramarginal Gyrus**	*	0.1/0.0	3.9 (-36, -52, 36)/-999.0 (0, 0, 0)
**Cingulate Gyrus**	31	0.1/0.1	3.7 (-7, -45, 40)/3.6 (9, -41, 34)
**Cuneus**	*	0.1/0.0	3.7 (-4, -93, 9)/-999.0 (0, 0, 0)
**Paracentral Lobule**	6	0.0/0.1	-999.0 (0, 0, 0)/3.5 (9, -29, 53)

ICGM10

**Area**	**Brodmann Area**	**volume (cc)**	**random effects: Max Value (x, y, z)**
**Middle Temporal Gyrus**	19, 21, 37, 39	3.0/4.1	7.7 (-45, -56, 25)/11.8 (43, -62, 5)
**Superior Temporal Gyrus**	22, 39	1.3/1.0	9.1 (-40, -56, 26)/5.0 (46, -54, 25)
**Sub-Gyral**	*	1.7/0.9	7.3 (-43, -54, -7)/8.7 (40, -61, 7)

**Cerebellar Tonsil**	*****	**1.9/1.3**	**8.4 (-12, -57, -41)/5.8 (4, -56, -41)**
**Inferior Temporal Gyrus**	**19, 20, 21, 37**	**0.8/0.7**	**6.3 (-53, -54, -6)/7.8 (43, -65, 2)**
*****	*****	**0.1/0.0**	**4.6 (-3, -79, -28)/-999.0 (0, 0, 0)**
**Thalamus**	*****	**2.9/1.1**	**7.5 (-7, -16, 9)/6.5 (4, -11, 5)**
**Middle Occipital Gyrus**	**19**	**0.1/0.3**	**4.8 (-50, -57, -6)/7.4 (40, -65, 6)**
**Extra-Nuclear**	*****	**1.7/1.4**	**5.0 (-15, 8, 13)/5.3 (15, 12, 5)**
**Lentiform Nucleus**	*****	**3.2/2.7**	**6.4 (-25, 2, 3)/7.2 (28, -10, 3)**
**Inferior Semi-Lunar Lobule**	*****	**2.8/1.5**	**7.2 (-9, -60, -41)/5.9 (10, -60, -42)**
**Caudate**	*****	**2.4/2.2**	**6.1 (-12, 5, 14)/7.0 (13, 16, 3)**
**Cuneus**	**7, 18, 19, 23, 30**	**1.6/0.4**	**6.9 (-19, -82, 26)/5.6 (22, -83, 22)**
**Angular Gyrus**	**39**	**0.8/0.5**	**6.9 (-43, -60, 32)/4.4 (46, -64, 32)**
**Supramarginal Gyrus**	**40**	**0.9/0.4**	**6.8 (-43, -53, 27)/4.6 (48, -51, 27)**
**Uvula**	*****	**0.9/0.4**	**6.2 (-9, -77, -33)/4.4 (24, -76, -26)**
**Inferior Parietal Lobule**	**39, 40**	**0.8/0.6**	**6.0 (-37, -30, 39)/5.7 (40, -56, 39)**
**Pyramis**	*****	**2.0/0.7**	**6.0 (-6, -79, -30)/4.3 (36, -70, -31)**
**Third Ventricle**	*****	**0.1/0.1**	**3.5 (-1, -17, 1)/5.9 (1, -9, 2)**
**Precuneus**	**19, 31, 39**	**0.7/0.2**	**5.7 (-22, -79, 27)/4.9 (15, -59, 38)**
**Culmen**	*****	**2.0/1.7**	**5.3 (-33, -40, -22)/4.3 (34, -41, -26)**
**Tuber**	*****	**0.6/0.4**	**5.1 (-30, -73, -29)/4.8 (28, -74, -28)**
**Fusiform Gyrus**	**20**	**0.4/0.1**	**5.0 (-43, -53, -11)/3.6 (56, -6, -26)**
**Pyramis of Vermis**	*****	**0.1/0.0**	**4.9 (-3, -76, -30)/-999.0 (0, 0, 0)**
**Insula**	**13**	**0.1/0.1**	**4.6 (-45, -37, 21)/3.8 (37, -4, -1)**
**Postcentral Gyrus**	**2**	**0.3/0.1**	**4.5 (-45, -26, 37)/3.8 (39, -36, 58)**
**Declive**	*****	**0.3/0.0**	**4.5 (-45, -52, -20)/-999.0 (0, 0, 0)**
**Middle Frontal Gyrus**	*****	**0.1/0.0**	**4.4 (-33, -1, 43)/-999.0 (0, 0, 0)**
**Parahippocampal Gyrus**	*****	**0.1/0.0**	**4.4 (-25, -38, 3)/-999.0 (0, 0, 0)**
**Superior Occipital Gyrus**	**39**	**0.1/0.0**	**3.8 (-33, -81, 32)/-999.0 (0, 0, 0)**
**Superior Parietal Lobule**	*****	**0.0/0.1**	**-999.0 (0, 0, 0)/3.8 (30, -53, 45)**
**Cingulate Gyrus**	**31**	**0.1/0.0**	**3.7 (-9, -43, 35)/-999.0 (0, 0, 0)**

ICWM1

**Area**	**Brodmann Area**	**volume (cc)**	**random effects: Max Value (x, y, z)**
*	*	0.1/0.1	4.1 (-65, -53, 14)/4.2 (64, -47, -14)
Inferior Temporal Gyrus	19, 20, 21, 37	3.1/3.5	9.9 (-53, -10, -31)/9.7 (40, -13, -31)
Superior Frontal Gyrus	8	0.0/0.8	-999.0 (0, 0, 0)/9.8 (39, 20, 49)
Middle Frontal Gyrus	6, 8	0.1/1.9	3.7 (-37, 16, 56)/8.5 (36, 20, 46)
Middle Temporal Gyrus	19, 20, 21, 22, 37, 38, 39	5.4/8.3	7.6 (-42, 1, -33)/8.2 (40, -3, -33)
Middle Occipital Gyrus	18, 19	0.0/2.0	-999.0 (0, 0, 0)/8.1 (34, -81, 15)
Supramarginal Gyrus	40	0.6/0.1	8.0 (-61, -42, 30)/3.6 (56, -55, 35)
Fusiform Gyrus	20, 36	2.0/1.0	7.8 (-56, -16, -26)/6.6 (40, -13, -26)
Uncus	20	0.1/0.2	4.9 (-37, -15, -28)/7.1 (37, -13, -28)
Sub-Gyral	20	1.7/2.2	5.8 (-45, -5, -21)/7.0 (27, -56, 40)
Inferior Parietal Lobule	40	0.5/0.2	6.8 (-64, -41, 27)/4.4 (49, -40, 45)
Cuneus	7, 18, 19	1.7/2.9	6.5 (-7, -75, 36)/6.1 (13, -89, 18)
Superior Temporal Gyrus	21, 22, 38	1.6/0.7	6.4 (-65, -50, 11)/5.6 (65, -4, -1)
Superior Parietal Lobule	7	0.5/0.7	5.6 (-30, -51, 55)/6.3 (27, -59, 43)
Precuneus	7, 31	2.9/2.2	6.0 (-13, -70, 27)/6.1 (24, -59, 40)
Postcentral Gyrus	2, 3, 5	1.0/0.2	5.5 (-64, -20, 29)/5.3 (31, -39, 67)
Precentral Gyrus	4	0.5/0.1	4.8 (-43, -15, 49)/4.5 (46, -14, 62)
Inferior Occipital Gyrus	*	0.0/0.1	-999.0 (0, 0, 0)/4.5 (39, -72, -3)
Angular Gyrus	*	0.0/0.1	-999.0 (0, 0, 0)/4.1 (33, -57, 35)
Inferior Frontal Gyrus	9	0.0/0.3	-999.0 (0, 0, 0)/4.0 (53, 14, 27)
Parahippocampal Gyrus	36	0.1/0.0	3.9 (-37, -22, -23)/-999.0 (0, 0, 0)
Extra-Nuclear	*	0.1/0.1	3.6 (-36, -5, 9)/3.9 (37, -3, 6)
Medial Frontal Gyrus	*	0.1/0.0	3.7 (-10, -7, 58)/-999.0 (0, 0, 0)
Lingual Gyrus	*	0.0/0.1	-999.0 (0, 0, 0)/3.6 (12, -89, -2)

ICWM3

Area	Brodmann Area	volume (cc)	random effects: Max Value (x, y, z)
Supramarginal Gyrus	40	1.3/1.9	5.8 (-49, -45, 31)/8.2 (56, -48, 34)
*	*	0.0/0.0	-999.0 (0, 0, 0)/-999.0 (0, 0, 0)
Cuneus	17, 18, 19, 23, 30	3.6/3.4	7.8 (-13, -71, 8)/7.1 (15, -67, 9)
Middle Temporal Gyrus	19, 20, 21, 37, 39	3.1/0.2	7.3 (-42, -77, 25)/4.2 (46, -71, 27)
Middle Frontal Gyrus	6, 8, 9, 11, 46	0.9/1.0	5.3 (-15, 48, -21)/7.2 (49, 34, 31)
Superior Frontal Gyrus	8, 9, 10, 11	3.6/4.2	6.3 (-13, 23, 46)/7.0 (22, 47, 32)
Inferior Parietal Lobule	7, 39, 40	2.6/3.1	6.0 (-31, -51, 55)/7.0 (43, -60, 44)
Sub-Gyral	*	3.1/0.6	7.0 (-34, -57, -6)/5.0 (27, -85, -2)
Lingual Gyrus	17, 18, 19	1.1/2.3	5.9 (-25, -93, -4)/6.6 (15, -84, 2)
Medial Frontal Gyrus	6, 9, 10, 11, 25	0.9/1.1	4.9 (-12, 37, 35)/6.3 (12, 3, 57)
Superior Parietal Lobule	7	0.9/0.3	6.0 (-31, -51, 48)/6.3 (42, -62, 49)
Superior Occipital Gyrus	19	0.4/0.0	6.2 (-45, -77, 27)/-999.0 (0, 0, 0)
Precuneus	7, 19, 31	1.2/0.2	6.1 (-13, -84, 39)/3.8 (25, -77, 43)
Inferior Occipital Gyrus	17, 18	1.4/0.1	6.0 (-34, -86, -2)/4.2 (30, -86, -5)
Cingulate Gyrus	9, 31, 32	0.5/2.2	5.3 (-7, -54, 26)/6.0 (10, 16, 36)
Posterior Cingulate	30, 31	0.2/0.8	4.5 (-9, -67, 10)/5.6 (18, -66, 12)
Angular Gyrus	39	0.4/0.0	5.5 (-43, -70, 30)/-999.0 (0, 0, 0)
Middle Occipital Gyrus	18, 19, 37	1.9/0.3	5.4 (-34, -86, 2)/4.5 (36, -84, 18)
Superior Temporal Gyrus	22, 39, 42	0.2/0.4	4.5 (-61, -53, 19)/5.1 (64, -47, 12)
Inferior Temporal Gyrus	20	0.6/0.1	5.1 (-58, -21, -20)/4.1 (50, -36, -18)
Uncus	20, 28, 36	0.3/0.0	5.0 (-28, -10, -30)/-999.0 (0, 0, 0)
Fusiform Gyrus	*	0.3/0.3	4.9 (-58, -19, -23)/4.5 (36, -50, -9)
Inferior Frontal Gyrus	11, 47	0.3/0.1	4.3 (-18, 37, -21)/3.9 (13, 37, -20)
Orbital Gyrus	11	0.2/0.1	4.0 (-10, 50, -21)/3.6 (13, 48, -23)
Anterior Cingulate	*	0.0/0.1	-999.0 (0, 0, 0)/3.7 (10, 32, 23)
Postcentral Gyrus	*	0.1/0.0	3.6 (-58, -16, 27)/-999.0 (0, 0, 0)

ICWM4

Area	Brodmann Area	volume (cc)	random effects: Max Value (x, y, z)
Postcentral Gyrus	2, 3, 7, 40	4.3/0.4	8.1 (-42, -27, 43)/4.2 (16, -48, 63)
*	*	0.0/0.0	-999.0 (0, 0, 0)/-999.0 (0, 0, 0)
Posterior Cingulate	23	0.4/0.4	7.0 (-4, -29, 22)/7.1 (4, -29, 22)
Extra-Nuclear	*	2.0/1.2	7.0 (-3, -25, 23)/6.3 (3, -31, 18)
Cingulate Gyrus	23, 24, 31, 32	7.8/2.9	6.8 (-12, 0, 37)/6.3 (3, -28, 26)
Sub-Gyral	*	6.0/0.3	6.8 (-34, -29, 41)/4.4 (33, -64, -6)
Inferior Parietal Lobule	2, 40	4.5/1.3	6.7 (-42, -30, 40)/5.9 (42, -49, 47)
Middle Frontal Gyrus	6, 8	2.0/0.6	6.4 (-36, 23, 43)/4.7 (27, 14, 46)
Supramarginal Gyrus	40	2.1/0.6	6.2 (-45, -42, 31)/5.1 (58, -48, 33)
Precuneus	7, 19, 31	0.6/0.4	6.2 (-12, -76, 45)/4.1 (13, -47, 41)
Precentral Gyrus	4	0.3/0.0	6.0 (-39, -24, 37)/-999.0 (0, 0, 0)
Superior Frontal Gyrus	8, 11	1.3/0.7	5.6 (-31, 60, -14)/5.2 (42, 17, 48)
Anterior Cingulate	24, 33	0.3/0.3	4.9 (-4, 16, 25)/5.0 (4, 17, 23)
Insula	13	0.3/0.0	4.4 (-42, -38, 21)/-999.0 (0, 0, 0)
Middle Temporal Gyrus	39	0.2/0.0	4.2 (-46, -74, 13)/-999.0 (0, 0, 0)
Fusiform Gyrus	*	0.0/0.1	-999.0 (0, 0, 0)/4.1 (45, -53, -11)
Paracentral Lobule	4	0.1/0.1	3.9 (-7, -38, 64)/4.0 (13, -32, 54)
Medial Frontal Gyrus	6	0.1/0.1	3.7 (-12, 45, 25)/3.9 (12, 4, 58)
Inferior Frontal Gyrus	47	0.0/0.1	-999.0 (0, 0, 0)/3.8 (37, 16, -10)
Superior Temporal Gyrus	13	0.2/0.0	3.7 (-49, -43, 21)/-999.0 (0, 0, 0)
Middle Occipital Gyrus	*	0.0/0.1	-999.0 (0, 0, 0)/3.7 (39, -88, 10)
Cerebellar Tonsil	*	0.0/0.1	-999.0 (0, 0, 0)/3.6 (50, -56, -35)

ICWM5

Area	Brodmann Area	volume (cc)	random effects: Max Value (x, y, z)
**Cerebellar Tonsil**	*	6.4/5.8	7.4 (-21, -57, -34)/6.8 (18, -61, -34)
*	*	3.3/2.7	7.0 (-21, -58, -30)/6.0 (18, -51, -30)
**Pyramis**	*	1.0/1.4	6.5 (-19, -64, -30)/6.0 (22, -61, -30)
**Inferior Semi-Lunar Lobule**	*	0.9/0.6	6.2 (-19, -64, -35)/5.3 (30, -64, -35)
**Superior Frontal Gyrus**	6, 8	0.4/0.3	5.2 (-10, -5, 67)/6.2 (37, 17, 50)
**Uvula**	*	0.3/0.8	4.7 (-16, -67, -34)/6.2 (15, -64, -33)
**Middle Frontal Gyrus**	6, 8, 9, 46	2.1/1.3	5.9 (-42, 12, 42)/5.5 (36, 17, 46)
**Inferior Frontal Gyrus**	45	0.1/0.4	4.4 (-49, 21, 21)/5.6 (52, 26, 19)
**Culmen**	*	0.6/0.8	5.1 (-24, -58, -26)/4.8 (10, -55, -22)
**Nodule**	*	0.1/0.3	5.1 (-15, -57, -29)/4.9 (13, -58, -29)
**Inferior Parietal Lobule**	*	0.0/0.4	-999.0 (0, 0, 0)/4.9 (33, -47, 56)
**Middle Occipital Gyrus**	19	0.1/0.0	4.8 (-50, -73, 1)/-999.0 (0, 0, 0)
**Fastigium**	*	0.3/0.3	4.7 (-7, -55, -20)/4.6 (7, -58, -22)
**Sub-Gyral**	*	0.1/0.1	4.7 (-45, 20, 21)/4.0 (45, 23, 18)
**Medial Frontal Gyrus**	6	0.1/0.0	4.6 (-13, -8, 67)/-999.0 (0, 0, 0)
**Cuneus**	19	0.0/0.1	-999.0 (0, 0, 0)/4.5 (28, -83, 33)
**Postcentral Gyrus**	5	0.0/0.1	-999.0 (0, 0, 0)/4.5 (36, -45, 59)
**Supramarginal Gyrus**	*	0.0/0.2	-999.0 (0, 0, 0)/4.5 (49, -43, 35)
**Tuber**	*	0.0/0.1	-999.0 (0, 0, 0)/4.2 (30, -58, -29)
**Declive**	*	0.0/0.2	-999.0 (0, 0, 0)/4.2 (15, -62, -22)
**Precuneus**	7	0.0/0.1	-999.0 (0, 0, 0)/3.9 (31, -47, 52)
**Inferior Occipital Gyrus**	*	0.1/0.0	3.8 (-37, -89, -2)/-999.0 (0, 0, 0)
**Inferior Temporal Gyrus**	*	0.1/0.0	3.8 (-48, -70, 1)/-999.0 (0, 0, 0)
**Precentral Gyrus**	*	0.1/0.0	3.8 (-34, -7, 58)/-999.0 (0, 0, 0)
**Superior Parietal Lobule**	7	0.0/0.1	-999.0 (0, 0, 0)/3.7 (33, -48, 60)
**Angular Gyrus**	*	0.0/0.1	-999.0 (0, 0, 0)/3.7 (49, -58, 35)
**Superior Temporal Gyrus**	39	0.1/0.0	3.6 (-56, -55, 28)/-999.0 (0, 0, 0)
**Fusiform Gyrus**	*	0.1/0.0	3.6 (-37, -19, -24)/-999.0 (0, 0, 0)

ICWM7

Area	Brodmann Area	volume (cc)	random effects: Max Value (x, y, z)
**Middle Frontal Gyrus**	**8, 9, 10, 11, 46, 47**	**7.0/9.7**	**7.3 (-48, 22, 40)/8.2 (24, 60, 8)**
**Superior Frontal Gyrus**	**6, 8, 9, 10, 11**	**4.6/8.8**	**6.0 (-18, 61, 22)/7.2 (21, 60, 5)**
**Inferior Frontal Gyrus**	**10, 45, 46, 47**	**2.2/2.7**	**7.1 (-49, 40, 4)/7.1 (46, 44, -1)**
*	*	**0.0/0.0**	**-999.0 (0, 0, 0)/-999.0 (0, 0, 0)**
**Sub-Gyral**	**20**	**1.9/2.2**	**5.1 (-40, -20, -22)/6.1 (40, 42, 5)**
**Fusiform Gyrus**	**20, 36**	**0.3/0.0**	**6.1 (-40, -17, -24)/-999.0 (0, 0, 0)**
**Inferior Temporal Gyrus**	**20, 21, 37**	**0.9/0.3**	**6.1 (-59, -46, -15)/4.1 (39, -12, -30)**
**Middle Temporal Gyrus**	**20, 21, 37, 39**	**2.2/0.5**	**5.9 (-56, -37, -12)/4.5 (68, -32, -5)**
**Medial Frontal Gyrus**	**10, 11**	**0.7/0.6**	**4.6 (-12, 43, 10)/5.5 (18, 63, 5)**
**Cuneus**	**18, 19**	**1.4/0.0**	**5.0 (-7, -92, 16)/-999.0 (0, 0, 0)**
**Superior Temporal Gyrus**	**22, 39**	**0.4/0.4**	**4.3 (-45, -54, 22)/4.9 (64, -46, 12)**
**Middle Occipital Gyrus**	**18**	**0.4/0.0**	**4.7 (-10, -90, 16)/-999.0 (0, 0, 0)**
**Precentral Gyrus**	**9**	**0.1/0.1**	**4.6 (-45, 22, 36)/3.8 (43, 21, 35)**
**Uncus**	**20**	**0.1/0.0**	**4.4 (-40, -14, -27)/-999.0 (0, 0, 0)**
**Anterior Cingulate**	**10, 32**	**0.5/0.0**	**4.3 (-15, 44, 7)/-999.0 (0, 0, 0)**
**Inferior Occipital Gyrus**	**18**	**0.1/0.0**	**4.0 (-34, -83, -3)/-999.0 (0, 0, 0)**
**Precuneus**	*	**0.0/0.1**	**-999.0 (0, 0, 0)/3.8 (22, -56, 48)**
**Postcentral Gyrus**	**5, 40**	**0.1/0.1**	**3.7 (-30, -41, 63)/3.6 (56, -28, 50)**
**Lingual Gyrus**	*	**0.0/0.1**	**-999.0 (0, 0, 0)/3.7 (24, -68, 5)**
**Inferior Parietal Lobule**	**40**	**0.0/0.1**	**-999.0 (0, 0, 0)/3.6 (45, -60, 47)**

ICWM8

Area	Brodmann Area	volume (cc)	random effects: Max Value (x, y, z)
**Postcentral Gyrus**	**1, 2, 3, 4, 40**	**3.7/1.3**	**8.5 (-36, -31, 57)/6.2 (39, -32, 62)**
**Medial Frontal Gyrus**	**6, 8, 32**	**0.2/2.0**	**3.9 (-7, -14, 67)/8.2 (15, 7, 51)**
**Middle Frontal Gyrus**	**6, 8, 9, 46**	**3.4/2.7**	**6.9 (-24, 27, 46)/7.5 (48, 35, 34)**

**Precentral Gyrus**	**4, 6, 9**	**2.4/2.7**	**7.2 (-33, -29, 54)/6.2 (59, -17, 40)**
*	*	**0.1/0.3**	**4.4 (-53, 29, 34)/4.9 (16, -64, -25)**
**Superior Frontal Gyrus**	**6, 8, 9, 10, 11**	**1.7/4.9**	**5.7 (-27, 27, 48)/7.0 (18, 10, 51)**
**Sub-Gyral**	**6**	**0.8/2.6**	**4.3 (-28, -28, 47)/6.7 (15, 10, 53)**
**Uncus**	**20**	**0.1/0.0**	**5.9 (-40, -14, -27)/-999.0 (0, 0, 0)**
**Middle Temporal Gyrus**	**21**	**1.8/0.3**	**5.8 (-56, -51, 5)/3.8 (52, -44, -7)**
**Pyramis**	*	**0.0/1.0**	**-999.0 (0, 0, 0)/5.4 (16, -67, -27)**
**Supramarginal Gyrus**	**40**	**0.4/0.0**	**5.3 (-59, -44, 31)/-999.0 (0, 0, 0)**
**Inferior Parietal Lobule**	**2, 40**	**0.9/0.3**	**5.1 (-50, -47, 45)/5.2 (64, -31, 29)**
**Fusiform Gyrus**	**20**	**0.1/0.0**	**5.1 (-40, -17, -24)/-999.0 (0, 0, 0)**
**Inferior Frontal Gyrus**	**9, 44**	**0.0/0.6**	**-999.0 (0, 0, 0)/5.1 (64, 12, 15)**
**Precuneus**	**7, 19**	**0.4/0.1**	**5.1 (-22, -75, 42)/4.3 (10, -72, 44)**
**Uvula**	*	**0.0/0.4**	**-999.0 (0, 0, 0)/5.0 (13, -67, -24)**
**Middle Occipital Gyrus**	*	**0.0/0.1**	**-999.0 (0, 0, 0)/4.5 (49, -74, 0)**
**Inferior Temporal Gyrus**	**20**	**0.3/0.1**	**4.4 (-40, -12, -30)/3.6 (53, -29, -17)**
**Cingulate Gyrus**	**24, 32**	**0.6/0.1**	**4.2 (-9, 6, 38)/4.3 (18, 4, 45)**
**Declive**	*	**0.0/0.3**	**-999.0 (0, 0, 0)/4.0 (13, -64, -22)**
**Cerebellar Tonsil**	*	**0.0/0.1**	**-999.0 (0, 0, 0)/3.9 (21, -64, -32)**
**Nodule**	*	**0.0/0.1**	**-999.0 (0, 0, 0)/3.9 (10, -64, -25)**
**Superior Parietal Lobule**	**7**	**0.0/0.1**	**-999.0 (0, 0, 0)/3.9 (28, -64, 53)**
**Superior Temporal Gyrus**	**22, 39**	**0.1/0.1**	**3.8 (-53, -53, 11)/3.8 (62, -52, 16)**
**Culmen**	*	**0.0/0.1**	**-999.0 (0, 0, 0)/3.7 (21, -61, -25)**

ICWM9

**Area**	**Brodmann Area**	**volume (cc)**	**random effects: Max Value (x, y, z)**
**Sub-Gyral**	**7, 10, 40**	**1.9/0.3**	**9.4 (-27, -50, 56)/4.2 (52, -30, -10)**
**Superior Frontal Gyrus**	**6, 8, 9, 10, 11**	**0.6/1.9**	**5.0 (-12, 56, 22)/9.0 (33, 47, 28)**
**Inferior Frontal Gyrus**	**10, 45, 46, 47**	**5.2/1.7**	**8.9 (-45, 41, 5)/5.9 (55, 30, 15)**
**Superior Parietal Lobule**	**7**	**0.9/0.1**	**8.7 (-28, -52, 59)/3.6 (36, -49, 59)**

**Precuneus**	**7**	**0.8/0.0**	**7.7 (-27, -48, 51)/-999.0 (0, 0, 0)**
**Middle Frontal Gyrus**	**8, 9, 10, 11, 46, 47**	**1.9/0.8**	**7.0 (-48, 45, -4)/7.6 (30, 46, 25)**
**Inferior Parietal Lobule**	**40**	**0.4/0.4**	**6.7 (-30, -50, 54)/5.2 (55, -37, 41)**
*	*	**0.1/0.3**	**3.5 (-25, -47, -30)/4.0 (18, 66, -13)**
**Middle Temporal Gyrus**	**19, 21, 22, 37, 38, 39**	**2.9/1.1**	**6.6 (-50, -75, 18)/5.6 (61, -44, 4)**
**Fusiform Gyrus**	**20**	**0.1/0.6**	**4.0 (-55, -17, -23)/5.9 (56, -20, -23)**
**Middle Occipital Gyrus**	**18, 19**	**1.0/0.0**	**5.1 (-33, -88, 10)/-999.0 (0, 0, 0)**
**Inferior Temporal Gyrus**	**19, 20, 37**	**1.0/1.4**	**4.9 (-56, -21, -17)/5.1 (56, -23, -20)**
**Angular Gyrus**	*	**0.1/0.0**	**4.9 (-50, -59, 35)/-999.0 (0, 0, 0)**
**Superior Temporal Gyrus**	**22**	**0.1/0.1**	**4.8 (-59, -51, 21)/3.9 (62, -43, 8)**
**Postcentral Gyrus**	**5**	**0.1/0.0**	**4.7 (-10, -43, 64)/-999.0 (0, 0, 0)**
**Supramarginal Gyrus**	**40**	**0.2/0.4**	**4.3 (-59, -54, 23)/4.6 (55, -38, 36)**
**Cuneus**	**18**	**0.5/0.0**	**4.6 (-21, -96, 2)/-999.0 (0, 0, 0)**
**Cerebellar Tonsil**	*	**0.6/0.2**	**4.4 (-46, -60, -32)/3.9 (24, -44, -31)**
**Paracentral Lobule**	**5**	**0.1/0.0**	**4.3 (-16, -42, 57)/-999.0 (0, 0, 0)**
**Pyramis**	*	**0.1/0.0**	**4.1 (-46, -64, -31)/-999.0 (0, 0, 0)**
**Anterior Cingulate**	**10**	**0.0/0.1**	**-999.0 (0, 0, 0)/3.9 (10, 48, -2)**
**Lingual Gyrus**	*	**0.1/0.0**	**3.9 (-19, -96, -5)/-999.0 (0, 0, 0)**
**Medial Frontal Gyrus**	*	**0.2/0.1**	**3.8 (-9, 2, 61)/3.5 (13, 1, 54)**

ICWM10

**Area**	**Brodmann Area**	**volume (cc)**	**random effects: Max Value (x, y, z)**
**Inferior Parietal Lobule**	**7, 39, 40**	**5.6/5.0**	**7.3 (-42, -47, 41)/7.8 (43, -58, 42)**
**Superior Parietal Lobule**	**7**	**0.2/0.2**	**5.6 (-40, -59, 51)/7.1 (42, -60, 50)**
**Supramarginal Gyrus**	**40**	**1.7/1.6**	**5.8 (-39, -45, 35)/6.7 (43, -45, 37)**
**Angular Gyrus**	**39**	**0.3/0.7**	**4.4 (-36, -54, 36)/6.5 (43, -58, 36)**
*	*	**0.8/0.6**	**4.2 (-21, -55, -29)/4.0 (28, -51, -30)**
**Extra-Nuclear**	*	**2.8/2.0**	**6.1 (-1, -30, 21)/6.0 (3, -30, 21)**
**Posterior Cingulate**	**23**	**0.1/0.3**	**5.8 (-4, -28, 24)/6.0 (6, -28, 24)**

**Cingulate Gyrus**	**23, 24, 31, 32**	**2.8/3.3**	**5.4 (0, -19, 26)/6.0 (3, -23, 26)**
**Sub-Gyral**	*****	**6.8/4.2**	**5.5 (-30, -59, 22)/5.0 (37, -46, 33)**
**Superior Temporal Gyrus**	**13, 22, 38, 39**	**1.8/1.3**	**4.7 (-37, -53, 22)/5.5 (45, -53, 12)**
**Cuneus**	**19**	**0.8/0.0**	**5.4 (-21, -87, 29)/-999.0 (0, 0, 0)**
**Lateral Ventricle**	*****	**0.5/0.4**	**5.1 (-12, -36, 17)/4.7 (13, -36, 17)**
**Middle Temporal Gyrus**	**19, 21, 39**	**2.0/2.2**	**5.0 (-34, -56, 22)/4.9 (45, -51, 9)**
**Cerebellar Tonsil**	*****	**1.7/2.7**	**4.3 (-22, -53, -31)/4.7 (31, -54, -36)**
**Middle Frontal Gyrus**	**10, 46**	**0.4/1.0**	**4.6 (-39, 47, 14)/4.7 (43, 42, 23)**
**Postcentral Gyrus**	**2, 3, 5**	**0.1/1.0**	**3.6 (-28, -30, 64)/4.7 (46, -29, 35)**
**Inferior Frontal Gyrus**	**13, 45, 47**	**0.0/0.2**	**-999.0 (0, 0, 0)/4.5 (37, 11, -12)**
**Superior Frontal Gyrus**	**10**	**0.3/0.1**	**4.3 (-13, 59, 18)/3.6 (39, 53, 14)**
**Insula**	**13**	**0.1/0.0**	**4.2 (-40, -41, 20)/-999.0 (0, 0, 0)**
**Precentral Gyrus**	**4**	**0.1/0.1**	**3.5 (-50, -11, 32)/4.1 (28, -29, 54)**
**Middle Occipital Gyrus**	*****	**0.0/0.1**	**-999.0 (0, 0, 0)/4.1 (34, -68, 16)**
**Lingual Gyrus**	*****	**0.1/0.1**	**4.0 (-15, -71, 5)/4.0 (18, -81, 3)**
**Precuneus**	**31, 39**	**0.1/0.1**	**3.8 (-39, -65, 35)/4.0 (21, -41, 30)**
**Pyramis**	*****	**0.2/0.0**	**3.9 (-21, -60, -30)/-999.0 (0, 0, 0)**
**Inferior Semi-Lunar Lobule**	*****	**0.1/0.0**	**3.7 (-18, -63, -35)/-999.0 (0, 0, 0)**
**Culmen**	*****	**0.1/0.0**	**3.6 (-22, -58, -26)/-999.0 (0, 0, 0)**
**Uvula**	*****	**0.1/0.0**	**3.5 (-16, -66, -32)/-999.0 (0, 0, 0)**

Supplementary Table S2, T2: MEDIATION ANALYSIS

Direct effects

						95% Confidence Interval	
		**Estimate**	**Std. Error**	**z-value**	**p**	**Lower**	**Upper**
CTQ-PhysAbuse	→ ZANaffectiveS	0.061	0.296	0.205	0.838	-0.519	0.641
CTQ-EmotAbuse	→ ZANaffectiveS	-0.240	0.185	-1.299	0.194	-0.603	0.122
CTQ-SexAbuse	→ ZANaffectiveS	0.291	0.139	2.093	0.036	0.018	0.563
CTQ-PhysNeglect	→ ZANaffectiveS	-0.191	0.288	-0.661	0.509	-0.756	0.375
CTQ-EmotNeglect	→ ZANaffectiveS	0.207	0.134	1.545	0.122	-0.056	0.470

Direct effects

						95% Confidence Interval	
		Estimate	Std. Error	z-value	p	Lower	Upper
CTQ-PhysAbuse	→ ZANcognS	0.159	0.169	0.945	0.345	-0.171	0.490
CTQ-EmotAbuse	→ ZANcognS	0.251	0.332	0.756	0.450	-0.400	0.901
CTQ-SexAbuse	→ ZANcognS	-0.012	0.133	-0.090	0.928	-0.273	0.249
CTQ-PhysNeglect	→ ZANcognS	-0.065	0.277	-0.234	0.815	-0.609	0.479
CTQ-EmotNeglect	→ ZANcognS	0.059	0.063	0.937	0.349	-0.064	0.182
CTQ-PhysAbuse	→ ZANimpulsS	-0.127	0.162	-0.783	0.433	-0.445	0.191
CTQ-EmotAbuse	→ ZANimpulsS	0.253	0.217	1.166	0.244	-0.172	0.678
CTQ-SexAbuse	→ ZANimpulsS	0.043	0.141	0.303	0.762	-0.234	0.320
CTQ-PhysNeglect	→ ZANimpulsS	0.231	0.214	1.083	0.279	-0.187	0.650
CTQ-EmotNeglect	→ ZANimpulsS	0.021	0.060	0.355	0.722	-0.096	0.138
CTQ-PhysAbuse	→ ZANinterpS	-0.352	0.182	-1.934	0.053	-0.708	0.005
CTQ-EmotAbuse	→ ZANinterpS	0.249	0.250	0.999	0.318	-0.240	0.739
CTQ-SexAbuse	→ ZANinterpS	0.251	0.139	1.811	0.070	-0.021	0.522
CTQ-PhysNeglect	→ ZANinterpS	-0.509	0.144	-3.540	< .001	-0.791	-0.227
CTQ-EmotNeglect	→ ZANinterpS	0.119	0.098	1.214	0.225	-0.073	0.312

Note. Robust standard errors, robust confidence intervals, ML estimator.

Indirect effects

							95% Confidence Interval	
			Estimate	Std. Error	z-value	p	Lower	Upper
CTQ-PhysAbuse	→ ICGM2	→ ZANaffectiveS	-0.242	0.174	-1.389	0.165	-0.584	0.100
CTQ-PhysAbuse	→ ICGM6	→ ZANaffectiveS	-0.167	0.195	-0.857	0.392	-0.548	0.215
CTQ-PhysAbuse	→ ICWM2	→ ZANaffectiveS	0.236	0.174	1.360	0.174	-0.104	0.577
CTQ-PhysAbuse	→ ICWM6	→ ZANaffectiveS	0.129	0.137	0.936	0.349	-0.141	0.398
CTQ-EmotAbuse	→ ICGM2	→ ZANaffectiveS	0.169	0.175	0.969	0.333	-0.173	0.512
CTQ-EmotAbuse	→ ICGM6	→ ZANaffectiveS	0.117	0.151	0.774	0.439	-0.179	0.413
CTQ-EmotAbuse	→ ICWM2	→ ZANaffectiveS	-0.116	0.134	-0.870	0.384	-0.378	0.146
CTQ-EmotAbuse	→ ICWM6	→ ZANaffectiveS	-0.021	0.056	-0.370	0.712	-0.131	0.090
CTQ-SexAbuse	→ ICGM2	→ ZANaffectiveS	-0.112	0.147	-0.757	0.449	-0.400	0.177
CTQ-SexAbuse	→ ICGM6	→ ZANaffectiveS	0.061	0.085	0.711	0.477	-0.107	0.228
CTQ-SexAbuse	→ ICWM2	→ ZANaffectiveS	-0.013	0.092	-0.139	0.889	-0.193	0.167
CTQ-SexAbuse	→ ICWM6	→ ZANaffectiveS	-0.095	0.102	-0.926	0.355	-0.295	0.106
CTQ-PhysNeglect	→ ICGM2	→ ZANaffectiveS	0.400	0.247	1.620	0.105	-0.084	0.884
CTQ-PhysNeglect	→ ICGM6	→ ZANaffectiveS	-0.181	0.222	-0.818	0.413	-0.616	0.253
CTQ-PhysNeglect	→ ICWM2	→ ZANaffectiveS	-0.147	0.122	-1.204	0.229	-0.386	0.092
CTQ-PhysNeglect	→ ICWM6	→ ZANaffectiveS	0.212	0.194	1.090	0.276	-0.169	0.592
CTQ-EmotNeglect	→ ICGM2	→ ZANaffectiveS	-0.222	0.114	-1.940	0.052	-0.445	0.002
CTQ-EmotNeglect	→ ICGM6	→ ZANaffectiveS	-0.005	0.031	-0.157	0.875	-0.066	0.056
CTQ-EmotNeglect	→ ICWM2	→ ZANaffectiveS	0.114	0.090	1.276	0.202	-0.061	0.290
CTQ-EmotNeglect	→ ICWM6	→ ZANaffectiveS	-0.012	0.043	-0.270	0.787	-0.095	0.072
CTQ-PhysAbuse	→ ICGM2	→ ZANcognS	-0.153	0.141	-1.084	0.278	-0.429	0.124
CTQ-PhysAbuse	→ ICGM6	→ ZANcognS	0.179	0.167	1.074	0.283	-0.148	0.506
CTQ-PhysAbuse	→ ICWM2	→ ZANcognS	0.232	0.139	1.676	0.094	-0.039	0.504
CTQ-PhysAbuse	→ ICWM6	→ ZANcognS	-0.148	0.164	-0.900	0.368	-0.469	0.174
CTQ-EmotAbuse	→ ICGM2	→ ZANcognS	0.107	0.121	0.887	0.375	-0.129	0.343
CTQ-EmotAbuse	→ ICGM6	→ ZANcognS	-0.126	0.091	-1.389	0.165	-0.303	0.052
CTQ-EmotAbuse	→ ICWM2	→ ZANcognS	-0.114	0.126	-0.904	0.366	-0.362	0.133
CTQ-EmotAbuse	→ ICWM6	→ ZANcognS	0.024	0.056	0.424	0.672	-0.087	0.134

Indirect effects

						95% Confidence Interval	
			Estimate	Std. Error	z-value	p	Lower	Upper
CTQ-SexAbuse	→ ICGM2	→ ZANcognS	-0.070	0.107	-0.659	0.510	-0.280	0.139
CTQ-SexAbuse	→ ICGM6	→ ZANcognS	-0.065	0.078	-0.838	0.402	-0.218	0.087
CTQ-SexAbuse	→ ICWM2	→ ZANcognS	-0.013	0.090	-0.140	0.889	-0.189	0.163
CTQ-SexAbuse	→ ICWM6	→ ZANcognS	0.109	0.124	0.877	0.380	-0.134	0.352
CTQ-PhysNeglect	→ ICGM2	→ ZANcognS	0.252	0.275	0.919	0.358	-0.286	0.791
CTQ-PhysNeglect	→ ICGM6	→ ZANcognS	0.195	0.147	1.329	0.184	-0.092	0.482
CTQ-PhysNeglect	→ ICWM2	→ ZANcognS	-0.145	0.122	-1.183	0.237	-0.384	0.095
CTQ-PhysNeglect	→ ICWM6	→ ZANcognS	-0.243	0.151	-1.612	0.107	-0.539	0.053
CTQ-EmotNeglect	→ ICGM2	→ ZANcognS	-0.140	0.109	-1.286	0.198	-0.353	0.073
CTQ-EmotNeglect	→ ICGM6	→ ZANcognS	0.005	0.033	0.160	0.873	-0.059	0.070
CTQ-EmotNeglect	→ ICWM2	→ ZANcognS	0.112	0.075	1.498	0.134	-0.035	0.260
CTQ-EmotNeglect	→ ICWM6	→ ZANcognS	0.013	0.051	0.259	0.795	-0.087	0.114
CTQ-PhysAbuse	→ ICGM2	→ ZANimpulsS	-0.328	0.203	-1.617	0.106	-0.724	0.069
CTQ-PhysAbuse	→ ICGM6	→ ZANimpulsS	0.122	0.148	0.822	0.411	-0.169	0.413
CTQ-PhysAbuse	→ ICWM2	→ ZANimpulsS	0.519	0.215	2.414	0.016	0.098	0.940
CTQ-PhysAbuse	→ ICWM6	→ ZANimpulsS	-0.174	0.173	-1.006	0.314	-0.513	0.165
CTQ-EmotAbuse	→ ICGM2	→ ZANimpulsS	0.229	0.232	0.990	0.322	-0.225	0.683
CTQ-EmotAbuse	→ ICGM6	→ ZANimpulsS	-0.086	0.088	-0.969	0.333	-0.259	0.088
CTQ-EmotAbuse	→ ICWM2	→ ZANimpulsS	-0.255	0.263	-0.969	0.333	-0.771	0.261
CTQ-EmotAbuse	→ ICWM6	→ ZANimpulsS	0.028	0.066	0.424	0.672	-0.102	0.158
CTQ-SexAbuse	→ ICGM2	→ ZANimpulsS	-0.151	0.197	-0.766	0.444	-0.537	0.235
CTQ-SexAbuse	→ ICGM6	→ ZANimpulsS	-0.044	0.066	-0.669	0.503	-0.174	0.086
CTQ-SexAbuse	→ ICWM2	→ ZANimpulsS	-0.028	0.200	-0.140	0.889	-0.421	0.365
CTQ-SexAbuse	→ ICWM6	→ ZANimpulsS	0.128	0.138	0.927	0.354	-0.143	0.399
CTQ-PhysNeglect	→ ICGM2	→ ZANimpulsS	0.541	0.308	1.755	0.079	-0.063	1.145
CTQ-PhysNeglect	→ ICGM6	→ ZANimpulsS	0.133	0.145	0.915	0.360	-0.152	0.417
CTQ-PhysNeglect	→ ICWM2	→ ZANimpulsS	-0.323	0.211	-1.531	0.126	-0.736	0.090
CTQ-PhysNeglect	→ ICWM6	→ ZANimpulsS	-0.286	0.168	-1.707	0.088	-0.615	0.043
CTQ-EmotNeglect	→ ICGM2	→ ZANimpulsS	-0.300	0.126	-2.370	0.018	-0.547	-0.052
CTQ-EmotNeglect	→ ICGM6	→ ZANimpulsS	0.004	0.022	0.161	0.872	-0.040	0.047
CTQ-EmotNeglect	→ ICWM2	→ ZANimpulsS	0.251	0.128	1.961	0.050	1.260e-4	0.502
CTQ-EmotNeglect	→ ICWM6	→ ZANimpulsS	0.016	0.060	0.263	0.793	-0.101	0.132
CTQ-PhysAbuse	→ ICGM2	→ ZANinterpS	-0.119	0.078	-1.520	0.129	-0.273	0.035
CTQ-PhysAbuse	→ ICGM6	→ ZANinterpS	0.579	0.277	2.092	0.036	0.036	1.121
CTQ-PhysAbuse	→ ICWM2	→ ZANinterpS	0.129	0.112	1.152	0.249	-0.091	0.349
CTQ-PhysAbuse	→ ICWM6	→ ZANinterpS	-0.285	0.255	-1.118	0.264	-0.785	0.215
CTQ-EmotAbuse	→ ICGM2	→ ZANinterpS	0.083	0.100	0.836	0.403	-0.112	0.279
CTQ-EmotAbuse	→ ICGM6	→ ZANinterpS	-0.406	0.224	-1.812	0.070	-0.845	0.033
CTQ-EmotAbuse	→ ICWM2	→ ZANinterpS	-0.063	0.080	-0.793	0.428	-0.220	0.093
CTQ-EmotAbuse	→ ICWM6	→ ZANinterpS	0.046	0.115	0.402	0.687	-0.179	0.271
CTQ-SexAbuse	→ ICGM2	→ ZANinterpS	-0.055	0.082	-0.666	0.505	-0.216	0.107
CTQ-SexAbuse	→ ICGM6	→ ZANinterpS	-0.211	0.195	-1.083	0.279	-0.592	0.171
CTQ-SexAbuse	→ ICWM2	→ ZANinterpS	-0.007	0.049	-0.141	0.887	-0.104	0.090
CTQ-SexAbuse	→ ICWM6	→ ZANinterpS	0.210	0.208	1.011	0.312	-0.197	0.617
CTQ-PhysNeglect	→ ICGM2	→ ZANinterpS	0.197	0.148	1.326	0.185	-0.094	0.488
CTQ-PhysNeglect	→ ICGM6	→ ZANinterpS	0.630	0.297	2.120	0.034	0.048	1.212
CTQ-PhysNeglect	→ ICWM2	→ ZANinterpS	-0.080	0.092	-0.874	0.382	-0.260	0.100
CTQ-PhysNeglect	→ ICWM6	→ ZANinterpS	-0.469	0.240	-1.952	0.051	-0.941	0.002
CTQ-EmotNeglect	→ ICGM2	→ ZANinterpS	-0.109	0.078	-1.398	0.162	-0.262	0.044
CTQ-EmotNeglect	→ ICGM6	→ ZANinterpS	0.017	0.105	0.163	0.871	-0.188	0.222
CTQ-EmotNeglect	→ ICWM2	→ ZANinterpS	0.062	0.068	0.925	0.355	-0.070	0.195

Indirect effects

					95% Confidence Interval	
	Estimate	Std. Error	z-value	p	Lower	Upper
CTQ-EmotNeglect → ICWM6 → ZANinterpS	0.026	0.096	0.266	0.790	-0.163	0.214

Note. Robust standard errors, robust confidence intervals, ML estimator.

R-Squared

	R^2
ZANaffectiveS	0.324
ZANcognS	0.306
ZANimpulsS	0.559
ZANinterpS	0.537
ICGM2	0.431
ICGM6	0.477
ICWM2	0.314
ICWM6	0.320

Supplementary Figure S1: Path plot

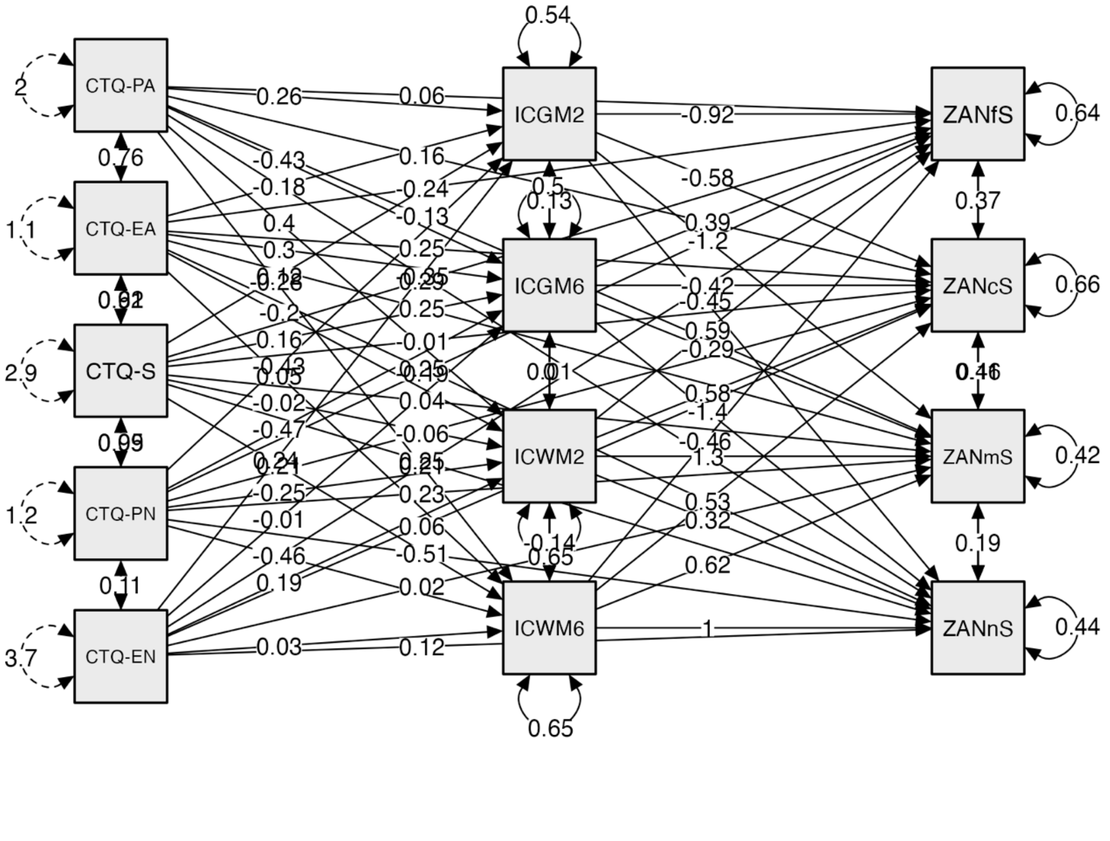

Supplement: Supplementary file 1 [file sensors-23-02862-s001.zip › sensors-2229571-supplementary.pdf]
